# Tuning the Fire: Context-Dependent Mitochondrial ROS Signaling, Mitohormesis, and Redox-Modulating Interventions

**DOI:** 10.3390/biom16060867

**Published:** 2026-06-12

**Authors:** Evelina Charidemou, Eleni Andreou, Christos Papaneophytou

**Affiliations:** Department of Life Sciences, School of Life and Health Sciences, University of Nicosia, Nicosia 2417, Cyprus; charidemou.e@unic.ac.cy (E.C.); andreou.el@unic.ac.cy (E.A.)

**Keywords:** mitohormesis, mitochondrial ROS, redox signaling, reverse electron transport, NRF2/KEAP1 pathway, redox buffering, phytochemicals, mitochondria-targeted therapeutics

## Abstract

Mitochondrial reactive oxygen species (mtROS) are central regulators of cellular function, yet their biological roles are often reduced to an oxidative-stress/antioxidant dichotomy. This review reframes mtROS through the concept of mitohormesis, in which outcomes are neither inherently harmful nor beneficial but are determined by a defined set of contextual variables. We present a mechanistic framework in which mtROS effects depend on chemical species identity, sub-mitochondrial site of production, temporal dynamics, redox-buffering capacity, and metabolic state; together, these variables determine whether mtROS promote adaptive eustress or pathological distress. We then show that, across polyphenols, isothiocyanates, terpenoids, alkaloids, and quinones, the biologically relevant effects of natural redox-modulating compounds are mediated less by direct radical scavenging than by pro-hormetic mechanisms, including mild electron transport chain perturbation, nuclear factor erythroid 2-related factor 2/Kelch-like ECH-associated protein 1 (NRF2/KEAP1) activation, modulation of mitochondrial membrane potential, mitochondrial quality control, and NAD^+^/NADPH regulation. Applying this framework to disease reveals strong tissue and state dependence: neurodegeneration favors buffering expansion and mitophagy; metabolic disease may benefit from exercise-mimetic and NRF2-activating strategies; cardiovascular disease illustrates mitohormesis through ischemic preconditioning and CoQ10 supplementation; and cancer requires distinction between prevention and therapy because redox buffering can either protect normal tissue or support tumor survival. Finally, we argue that the failure of non-specific antioxidant supplementation is mechanistically predictable and propose context-aware, biomarker-guided, temporally optimized, and compartment-targeted redox interventions as a more rational translational path.

## 1. Introduction

Mitochondria are double-membrane-bound organelles that contain their own genomes and are present in nearly all known eukaryotic cells, from single-celled organisms to humans [1]. Through the electron transport chain (ETC) and oxidative phosphorylation (OxPhos), mitochondria couple nutrient oxidation to ATP synthesis, meeting acute and chronic bioenergetic demands [2]. Beyond this canonical “powerhouse” function, they also serve as biosynthetic and signaling hubs, producing metabolites, lipids, amino acids, nucleotides, and essential cofactors such as heme and iron–sulfur clusters [3,4]. This metabolic versatility, together with a membrane-rich architecture and a redox-active proteome, places mitochondria at the intersection of energy transduction, metabolic rewiring, and cellular signaling.

Over the past three decades, mitochondria have also been recognized as functionally heterogeneous organelles whose spatial organization and bioenergetic properties are dynamically remodeled in response to cellular demands and stress [5,6]. Within individual cells, mitochondrial subpopulations can meet compartment-specific bioenergetic demands [7]. This heterogeneity is maintained by mitochondrial dynamics, including fission, fusion, mitophagy, and intracellular transport [8]. Disruption of these processes alters mitochondrial function, affects cell fate, and contributes to a wide range of pathologies, including neurodegenerative, metabolic, and cardiovascular diseases, as well as cancers [9]. The concept of a mitochondrial information processing system (MIPS) has been proposed to formalize this integrative role, building on earlier recognition of mitochondria as central regulators of cell fate, including apoptosis mediated by cytochrome c (cytc) release [10,11].

A central, and historically underappreciated, output of mitochondrial metabolism is the regulated generation of reactive oxygen species (ROS) [12]. ROS comprise both free radicals, such as superoxide $\left(O_{2}^{\cdot -}\right)$ and hydroxyl radical (HO˙), and non-radical oxidants, such as hydrogen peroxide (H_2_O_2_) [13]. Although ROS are also generated by other cellular systems, including NADPH oxidases, xanthine oxidase, myeloperoxidase, iron- and copper-containing proteins, and cytochrome P450 enzymes [14], mitochondrial ROS (mtROS) arise primarily from defined redox centers within the ETC and, in a context-dependent manner, from other mitochondrial enzymes [15]. Mitochondria are therefore widely regarded as a major source of intracellular ROS, particularly under conditions of elevated membrane potential and electron backpressure [3]. Early estimates suggest that ~0.1–2% of the oxygen consumed is partially reduced to superoxide, although these values vary with metabolic state [16].

The biological meaning of mtROS depends on species identity, sub-mitochondrial site of production, stimulus intensity and duration, and local redox-buffering capacity [17,18]. Excessive or sustained mtROS can cause macromolecular damage and drive pathology, whereas transient mtROS signals regulate immune responses, cell-cycle progression, hypoxic adaptation, and stress-resistance pathways [19]. For example, mtROS can increase under hypoxic conditions and contribute to oxygen sensing by stabilizing hypoxia-inducible factor (HIF) by inhibiting prolyl hydroxylases [20,21]. This context dependence underlies mitohormesis, a response in which transient or low-level mitochondrial stress activates adaptive pathways that enhance cellular fitness, whereas sustained or excessive stress promotes dysfunction [22]. Perturbations such as nutrient limitation, exercise, toxin exposure, or genetic stress can trigger coordinated responses that restore metabolic, proteostatic, and redox homeostasis, with adaptive effects that may persist beyond the initiating stimulus [23]. These responses involve mitonuclear communication via ROS and TCA cycle metabolites, proteotoxic signaling via the mitochondrial unfolded protein response (UPR^mt^), and secreted mitokines such as fibroblast growth factor 21 (FGF21) and growth differentiation factor 15 (GDF15) [24].

Because ATP and TCA cycle intermediates regulate cytosolic pathways that control epigenetics, ion flux, and inflammation, shifts in mitohormetic balance can influence the progression of metabolic, cardiovascular, and neurological diseases [25]. Notably, many natural biomolecules and phytochemicals historically classified as “antioxidants,” including polyphenols, flavonoids, and terpenoids, act in a context-dependent manner via pro-oxidative, mitochondria-engaged mechanisms. Rather than directly scavenging radicals, these compounds can transiently increase mtROS or modulate electron flow, thereby activating adaptive pathways such as the nuclear factor erythroid 2-related factor 2/Kelch-like ECH-associated protein 1 (NRF2/KEAP1) axis, AMPK signaling, and sirtuin-dependent regulation [26,27].

This mechanistic reframing has significant translational implications. It helps explain why high-dose, non-specific antioxidant supplementation has largely failed in clinical trials and suggests that context-aware, pro-hormetic modulation of mtROS is a more rational therapeutic strategy [28,29]. In this review, we revisit mitohormesis through a mechanistic lens, emphasizing mtROS as tunable, context-dependent signals. We integrate the molecular determinants that distinguish protective from pathological ROS signaling, analyze how natural compounds modulate mitochondrial redox biology, and discuss implications for disease prevention and therapy.

## 2. Mitohormesis: Concept and Mechanistic Framework

Mitochondria occupy a central position at the intersection of energy metabolism and cellular signaling, and transient increases in mtROS can trigger adaptive responses that enhance cellular stress resistance [10]. The term mitohormesis captures this relationship and situates it within the broader conceptual framework of hormesis in toxicology and physiology [30]. In its general form, hormesis describes a biphasic rather than linear dose–response relationship, in which low-level exposure elicits qualitatively different, often beneficial, effects compared with high-dose exposure [31]. Applied to mitochondria, this framework predicts that mild perturbations of mitochondrial function, whether metabolic, pharmacological, or environmental, can activate conserved stress-response programs whose protective effects persist beyond the initiating stimulus, leaving cells and tissues in a more resilient state [32]. Understanding how this process is regulated has implications for disease susceptibility and provides a unifying perspective on biological aging.

The relevance of mitohormesis is particularly evident in aging and age-related disease [33]. Aging is increasingly conceptualized as a progressive decline in physiological function that increases vulnerability to chronic conditions and shortens healthspan, the period of life free from substantial disease burden [34,35]. Although life expectancy has increased markedly over the past century, healthspan has not kept pace; one analysis of the Global Burden of Disease Study 2017 attributed 51.3% of the total adult disease burden worldwide to 92 age-related conditions [35,36]. Within the hallmarks of aging, mitochondrial dysfunction plays a prominent cross-cutting role, recurring across neurodegeneration, cardiometabolic disease, and cancer [37]. Preserving mitochondrial function is therefore central to physiological integrity, and its progressive decline is closely associated with reduced resilience and increased morbidity [38]. Against this backdrop, the central question of mitohormesis, namely how mild mitochondrial stress can be protective rather than harmful, is directly relevant to strategies aimed at extending healthspan rather than simply prolonging survival [39].

The following subsections trace the conceptual evolution of mitohormesis, from early free-radical theories to current mechanistic models, and define the contextual parameters that determine whether a given mtROS signal results in adaptation or injury.

### 2.1. From the Free-Radical Theory of Aging to Mitohormesis

The link between mitochondrial electron transport and ROS generation placed mitochondria at the center of early theories linking oxidative chemistry to biological aging [40]. In 1956, Denham Harman proposed that endogenous free radicals, produced as byproducts of normal metabolism, progressively damage cellular macromolecules and drive aging [41]. He later refined this idea by identifying the mitochondrial electron transport chain as a principal intracellular source of these species, thereby giving rise to the mitochondrial free-radical theory of aging [42]. Together with the formalization of “oxidative stress” as an imbalance between oxidant production and antioxidant defense [43], these ideas shaped a paradigm in which mitochondrial ROS were viewed primarily as harmful byproducts of aerobic metabolism [44].

Several findings challenged this damage-centric model. Large-scale antioxidant supplementation trials generally failed to demonstrate consistent benefits for age-related outcomes, and some interventions were associated with adverse effects in specific contexts [45,46,47,48]. Genetic studies in model organisms also yielded paradoxical results: certain long-lived mutants in *Caenorhabditis elegans* and *Drosophila melanogaster* exhibited elevated, rather than reduced, ROS levels, and mild respiratory perturbation, including partial knockdown of ETC components or altered coupling efficiency, extended lifespan in a ROS-dependent manner [49,50]. Similarly, exercise, one of the most robust promoters of healthspan, induces transient mitochondrial $O_{2}^{\cdot -}$/H_2_O_2_ signaling, and some exercise benefits are blunted by concurrent antioxidant supplementation [51]. Collectively, these observations indicate that mitochondrial oxidants can carry signaling information that may be lost when ROS are indiscriminately quenched [52].

These observations catalyzed a conceptual shift toward mitohormesis, in which mild, transient mitochondrial stress promotes adaptive remodeling rather than damage. The term “mitohormesis” emerged in the mid-2000s and was later developed into a framework linking transient increases in mtROS to stress-response activation and lifespan extension [27,53]. In parallel, redox biology refined oxidative-stress terminology by distinguishing oxidative eustress, physiological redox signaling that supports homeostasis and adaptation, from oxidative distress, oxidant exposure that exceeds buffering capacity and drives damage [54]. Thus, mitohormesis is best understood as a biphasic, context-dependent relationship between mtROS and biological outcome, rather than as a simple assertion that ROS are either beneficial or harmful [39,55].

### 2.2. Defining Features of the Mitohormetic Response

**Figure 1:**
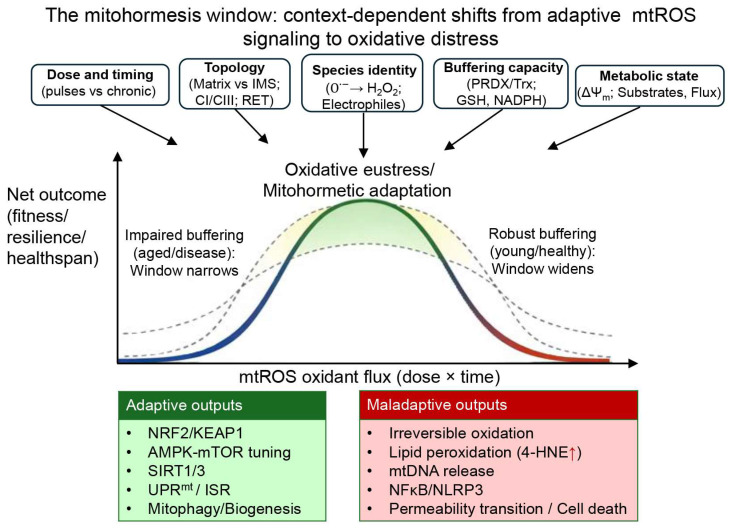
The mitohormesis window: context-dependent decoding of mtROS signals. mtROS elicit biphasic biological effects: low-to-moderate, typically transient oxidant flux promotes oxidative eustress and adaptive remodeling, whereas excessive or sustained mtROS production drives oxidative distress and pathology. The position and width of the mitohormetic window are shifted by interacting contextual determinants, including oxidant dose and temporal pattern; ROS identity, such as $O_{2}^{\cdot -}$, $O_{2}^{\cdot -}$ derived H_2_O_2_, and lipid-derived electrophiles; sub-mitochondrial topology of production, including matrix- versus intermembrane-space-facing sites at Complexes I and III and RET-associated signals; cellular redox-buffering capacity, including PRDX/Trx and glutathione systems and NADPH supply; and metabolic state, including ΔΨm, substrate availability, and respiratory flux. Adaptive outputs include NRF2 activation via KEAP1 modification, AMPK–mTOR tuning, sirtuin-linked programs, UPR^mt^/ISR activation, and mitophagy/biogenesis, whereas distress is associated with irreversible oxidation, excessive lipid peroxidation, inflammatory amplification through NF-κB/NLRP3, permeability transition, and cell death. Abbreviations: 4-HNE, 4-hydroxynonenal; AMPK, AMP-activated protein kinase; ATP, adenosine triphosphate; CI, Complex I; CIII, Complex III; ETC, electron transport chain; GSH, reduced glutathione; H_2_O_2_, hydrogen peroxide; IMM, inner mitochondrial membrane; IMS, intermembrane space; ISR, integrated stress response; KEAP1, Kelch-like ECH-associated protein 1; mTOR, mechanistic target of rapamycin; mtDNA, mitochondrial DNA; mtROS, mitochondrial reactive oxygen species; NADPH, reduced nicotinamide adenine dinucleotide phosphate; NF-κB, nuclear factor kappa-light-chain-enhancer of activated B cells; NLRP3, NOD-, LRR-, and pyrin domain-containing protein 3; NRF2, nuclear factor erythroid 2-related factor 2; $O_{2}^{\cdot -}$, superoxide anion radical; PRDX, peroxiredoxin; RET, reverse electron transport; SIRT1/3, sirtuin 1 and sirtuin 3; Trx, thioredoxin; UPRmt, mitochondrial unfolded protein response; ΔΨm, mitochondrial membrane potential. Symbol ↑ indicates an increase.

The mitohormetic response is defined by a set of interrelated features (Figure 1) that collectively distinguish it from both simple stress recovery and unchecked oxidative damage [33]. Understanding these features is essential for explaining why a given mtROS signal induces adaptation in one experimental or clinical context but contributes to pathology in another.

#### 2.2.1. Biphasic Dose–Response and the Eustress–Distress Threshold

A hallmark of mitohormesis is a biphasic, or inverted-U (Figure 1), relationship between mtROS levels and biological outcomes [16]. At low to moderate levels, mtROS engage protective gene programs and enhance cellular fitness; beyond a context-specific threshold, the same species can overwhelm buffering systems and promote macromolecular damage [16]. Importantly, this threshold is not fixed: it shifts with cellular energetic state, antioxidant reserve, and the sub-mitochondrial site of ROS production. A physiologically intuitive example involves mitochondrial membrane potential (ΔΨm): when ATP demand is high, proton flux through ATP synthase increases, ΔΨm decreases, and mtROS production often remains within a signaling-competent range. When ATP demand drops, ΔΨm can rise, increasing electron backpressure and favoring ROS generation at Complexes I and III, potentially shifting signaling toward oxidative distress [56]. Mild mitochondrial uncoupling, whether pharmacological or endogenous, can lower ΔΨm and attenuate excessive ROS formation while preserving ATP output, thereby widening the hormetic window [57].

#### 2.2.2. Temporal Dynamics: Transient Pulses Versus Chronic Elevation

The duration and temporal pattern of mtROS exposure are as important as amplitude in determining outcomes. Transient or pulsatile elevations in mtROS, such as those induced by exercise, brief ischemic episodes, or acute phytochemical exposure, can engage adaptive transcriptional programs and then resolve, allowing the cell to return to baseline with an enhanced protective repertoire [58]. By contrast, chronic or continuous mtROS elevation can sustain redox modifications beyond the reversible range, promote irreversible oxidation of catalytic cysteines, and bias pathways such as NF-κB and the NLRP3 inflammasome toward persistent inflammatory activation [52]. This temporal distinction helps reconcile otherwise paradoxical observations: intermittent mtROS signaling during exercise supports healthspan, whereas sustained mtROS associated with chronic mitochondrial dysfunction accelerates age-related decline; similarly, ischemic preconditioning can be protective, whereas prolonged ischemia–reperfusion promotes cardiomyocyte death [52].

#### 2.2.3. Site Specificity of mtROS Production

The biological meaning of an mtROS signal depends on where it originates within the mitochondrion. Complex III-derived ROS released into the intermembrane space (IMS) can stabilize HIF-1α, linking ETC redox state to hypoxic adaptation. In this context, HIF-1α refers primarily to the cytosolic/nuclear transcription factor regulated by prolyl hydroxylase-dependent degradation, rather than specifically to an outer mitochondrial membrane-associated pool [12]. Within Complex I, electrons can leak at functionally distinct sites: the flavin site (I_F_) and the ubiquinone-binding site (I_Q_). ROS generated at I_F_ are associated with age-related oxidative damage under conditions of NADH accumulation, whereas I_Q_-derived ROS generated during reverse electron transport (RET) have been implicated in stress adaptation, myotube differentiation, macrophage reprogramming, and oxygen sensing [59,60]. This intra-complex duality highlights a key principle: mitohormesis is not simply about “more or less” ROS, but about the appropriate species, at the appropriate site, for the appropriate duration.

#### 2.2.4. Mitonuclear Communication

Because the mitochondrial genome encodes only 13 electron transport chain subunits, the vast majority of the mitochondrial proteome is encoded by the nuclear genome. Adaptive responses to mitochondrial stress therefore require bidirectional mitonuclear communication, including nuclear control of mitochondrial protein expression and retrograde signaling from mitochondria to the nucleus [61]. Retrograde signals include H_2_O_2_ and other redox cues, TCA-cycle metabolites such as α-ketoglutarate, succinate, fumarate, and acetyl-CoA, proteotoxic cues that activate the UPR^mt^, and mtDNA release that engages innate immune pathways such as cGAS–STING [62,63,64,65]. Together, these signals translate localized mitochondrial perturbations into coordinated transcriptional responses that rewire cellular metabolism, proteostasis, redox buffering, and inflammatory tone.

#### 2.2.5. Activation of Conserved Adaptive Programs

Mitonuclear signals converge on conserved stress-response pathways that mediate the protective arm of mitohormesis. Key nodes include NRF2/KEAP1 signaling, which expands antioxidant and detoxification capacity [66], AMPK signaling, which promotes catabolism, autophagy, and mitochondrial biogenesis [67], sirtuin-dependent programs, which couple NAD^+^ availability to metabolic and longevity-associated transcriptional networks [68,69], UPR^mt^/ISR signaling, which restores mitochondrial proteostasis and is linked to lifespan extension in *C. elegans* [70,71]; and PINK1/Parkin-mediated mitophagy, which removes damaged mitochondria before they become persistent sources of pathological ROS [72]. Crosstalk among energy-sensing, redox, and quality-control programs forms an integrated adaptive network whose collective output exceeds that of individual components [73,74].

#### 2.2.6. Persistence of Protective Effects Beyond the Stimulus

A defining and clinically significant feature of mitohormesis is that adaptive benefits can outlast the initiating stress. Once activated, transcriptional programs driven by NRF2, FOXO factors, PGC-1α, and UPR^mt^ effectors remodel the proteome and metabolome over hours to days, producing durable changes in antioxidant capacity, mitochondrial mass and efficiency, proteostasis, and inflammatory tone. Epigenetic modifications supported by mitochondria-derived metabolites such as acetyl-CoA and α-ketoglutarate may further stabilize these adaptive states [63,75]. This persistence distinguishes mitohormesis from transient homeostatic buffering and provides a mechanistic basis for long-term phenotypes such as exercise-induced metabolic fitness, ischemic preconditioning memory, and lifespan extension in genetic or dietary models of mild mitochondrial stress [76].

#### 2.2.7. Non-Cell-Autonomous Signaling: Mitokines and Systemic Hormesis

Mitohormetic responses can extend beyond the stressed cell or tissue through endocrine-like communication [40]. Mitochondrial stress can induce circulating factors, or mitokines, that convey adaptive signals to distant organs. The best-characterized mammalian examples include FGF21 and GDF15, which are induced by mitochondrial stress and regulate systemic metabolism, stress adaptation, and inflammatory tone [77,78]. In *C. elegans*, neuron-specific mitochondrial perturbation can activate UPR^mt^ signaling in distal intestinal cells, providing genetic evidence for organism-wide coordination of mitohormetic responses [79,80]. Mitochondrial-derived peptides such as humanin and MOTS-c may provide an additional route through which the mitochondrial genome contributes to inter-organ communication [81].

Mechanistically, these factors act through distinct receptor and signaling axes: FGF21 signals primarily through FGFR1c/β-Klotho complexes to regulate systemic glucose, lipid, and energy metabolism, whereas GDF15 signals through the GFRAL/RET receptor complex in the hindbrain to coordinate appetite, stress responses, and systemic metabolic adaptation. Mitochondrial-derived peptides such as humanin and MOTS-c have been linked to cytoprotective and metabolic signaling, including pathways involving IGF-1, mTOR, AMPK, and inflammatory regulation, depending on cellular context [77,78,81].

This systemic dimension has important implications for natural-biomolecule interventions. A compound that triggers mild mitochondrial stress at a primary exposure site, such as the gut epithelium after oral ingestion, could confer broader effects through mitokines or other endocrine-like mediators, even when systemic distribution of the parent compound is limited. This concept overlaps with xenohormesis and broadens the therapeutic logic of mitohormesis-based strategies.

#### 2.2.8. An Integrated Model of Mitohormetic Signaling

Together, the features described above define a mitochondrial redox-signaling circuit in which mtROS generated at defined ETC sites are converted into compartment-specific H_2_O_2_ signals, decoded by redox relays and metabolic sensors, and transmitted to cytosolic and nuclear adaptive pathways. Matrix-directed signals, including those produced during RET at Complex I, preferentially engage PRDX3/Trx2-dependent buffering, NAD^+^/sirtuin-linked programs, UPR^mt^/ISR activation, and mitochondrial quality-control pathways such as PINK1/Parkin-mediated mitophagy. In contrast, IMS-directed signals, particularly from Complex III, are positioned to influence cytosolic redox-sensitive pathways, including HIF-1α stabilization and NRF2/KEAP1-linked stress responses, while mitochondrial bioenergetic changes can independently converge on AMPK-dependent energy sensing. Thus, mitochondrial regulation of NRF2 is primarily mediated by redox/electrophilic signaling, whereas mitochondrial regulation of AMPK is primarily mediated by changes in cellular energy state and bioenergetic stress [62,67].

Matrix NAD^+^/NADH status further links mitochondrial function to longevity-associated pathways by regulating mitochondrial sirtuins, influencing metabolite-dependent retrograde signaling, and contributing to integrated cellular NAD^+^ metabolism rather than acting as a freely diffusible signal between compartments [63,68,69].

These local responses are integrated with anterograde nuclear control of the mitochondrial proteome, because most mitochondrial proteins are nuclear-encoded and require coordinated transcription, translation, import, and assembly. When sustained or amplified, mitochondrial stress responses can also propagate systemically through mitokines such as FGF21 and GDF15, as well as mitochondrial-derived peptides such as humanin and MOTS-c. Thus, mitohormesis should be viewed not as a single linear pathway but as an integrated redox–metabolic communication network linking ETC topology, buffering capacity, nuclear transcription, mitochondrial quality control, and organismal adaptation.

## 3. Molecular Determinants of Context-Dependent mtROS Signaling

Four interdependent factors determine the biological meaning of an mtROS signal: (i) the chemical identity and reactivity of the species produced, which define diffusion range and target selectivity; (ii) the sub-mitochondrial site of origin, which determines whether ROS are generated toward the matrix or intermembrane space and therefore which downstream targets are accessible; (iii) the mode of electron flow, especially reverse electron transport (RET), which generates functionally distinct ROS signals; and (iv) redox-buffering capacity, which shapes signal amplitude, duration, and spatial reach, thereby separating eustress from distress [82,83]. These factors are continuously tuned by metabolic conditions such as substrate supply, respiratory flux, and membrane potential, which modulate mtROS production and decoding in real time. Their interactions create a dynamic signaling landscape in which relatively small changes can shift mtROS from protective adaptation to pathological injury.

### 3.1. Chemical Identity and Signaling Competence of mtROS

The term mtROS encompasses several chemically distinct species, each with characteristic reactivity, half-life, diffusion range, and capacity for selective target engagement. These differences are consequential: the identity of the species produced determines which molecular targets are modified, over what spatial scale, and with what degree of reversibility—properties that collectively define signaling competence [84]. Table 1 summarizes the key physicochemical and signaling features that determine whether a mitochondrial oxidant functions primarily as a compartment-restricted intermediate, a diffusible redox signal, or a mediator of oxidative damage.

The subsections below briefly expand on the key physicochemical features of major mtROS species and explain how these properties constrain their roles in redox signaling.

#### 3.1.1. Superoxide: The Proximal Mitochondrial ROS

Superoxide is the proximal ROS generated within mitochondria, formed by one-electron reduction of molecular oxygen at defined redox centers in the electron transport chain (ETC) [93]. At physiological pH, superoxide exists predominantly in its anionic form ($O_{2}^{\cdot -}$) rather than as its protonated conjugate acid, the hydroperoxyl radical ($HO_{2}^{\cdot }$; pKₐ ~4.8) [94]. This negative charge limits membrane permeability, confining $O_{2}^{\cdot -}$ largely to the compartment in which it is produced—the matrix or the intermembrane space (IMS) [95]. Functionally, superoxide reacts efficiently with iron–sulfur cluster enzymes (e.g., aconitase), potentially mobilizing catalytic iron and promoting downstream Fenton chemistry, but it is comparatively ineffective at directly oxidizing most protein thiols under physiological conditions [96,97]. Given its short lifetime and compartmental restriction, $O_{2}^{\cdot -}$ acts primarily as a local intermediate with major signaling relevance is as the precursor of H_2_O_2_ via dismutation [98].

#### 3.1.2. Superoxide Dismutases: A Gatekeeping Conversion Step

The conversion of superoxide to hydrogen peroxide is catalyzed by superoxide dismutases (SODs), a rapid, compartment-specific reaction. In mitochondria, SOD2 (MnSOD) converts matrix-derived $O_{2}^{\cdot -}$ to H_2_O_2_, whereas SOD1 (Cu/ZnSOD), present in the IMS and cytosol, performs the same reaction in those compartments [99,100]. This spatial organization effectively directs oxidant signaling: matrix H_2_O_2_ has privileged access to matrix redox circuitry such as PRDX3/Trx2, while IMS-derived H_2_O_2_ is positioned to communicate with the cytosol through outer-membrane permeability pathways, including VDAC, and facilitated diffusion. Thus, SOD activity is not merely detoxification; it is a signal-conversion and signal-partitioning step that channels superoxide into compartment-specific H_2_O_2_ signals [101].

#### 3.1.3. Hydrogen Peroxide: The Principal Signaling Species

Among mitochondria-derived oxidants, H_2_O_2_ is widely regarded as the primary information-carrying molecule because it is sufficiently stable to diffuse over short distances, can traverse membranes (often facilitated by aquaporins such as AQP8), and reacts preferentially with a defined subset of redox-sensitive protein thiols rather than indiscriminately with all biomolecules [102]. Its effective lifetime (milliseconds to seconds, depending on local peroxidase activity) allows H_2_O_2_ to engage signaling networks and to interface with redox relay systems that convert oxidant flux into selective protein modifications [103].

#### 3.1.4. The Cysteine Oxidation Hierarchy and the Eustress–Distress Boundary

The signaling specificity of H_2_O_2_ rests on cysteine chemistry. Most cysteine thiols (pKa ~8–9) are protonated at physiological pH and react slowly with H_2_O_2_. In contrast, a functionally important subset of cysteines resides in microenvironments that stabilize the thiolate anion (Cys–S^−^; often with pK_a_ ~5–6), enabling much faster reaction with H_2_O_2_ and creating selective redox “switches” [104]. Oxidation proceeds through a hierarchy with increasing irreversibility: sulfenic acid (Cys–SOH) and disulfides (Cys–S–S–Cys) are typically reversible and associated with signaling, whereas further oxidation to sulfinic (Cys–SO_2_H) and sulfonic (Cys–SO_3_H) states marks a transition toward distress and damage (with the notable exception that sulfiredoxin (Srx) can reverse sulfinylation of 2-Cys peroxiredoxins [105]. This chemistry provides a molecular basis for the mitohormetic threshold: transient H_2_O_2_ pulses favor reversible modifications, whereas sustained flux drives irreversible oxidation and loss of function. Canonical redox-regulated targets include protein tyrosine phosphatases (e.g., PTP1B, PTEN), KEAP1 (Nrf2 regulation), and ASK1 (Trx-dependent control) [106].

#### 3.1.5. Redox Relays: Peroxiredoxins as Signal Transducers

A common objection to H_2_O_2_ signaling is the “kinetic paradox”: peroxiredoxins and glutathione peroxidases react with H_2_O_2_ far faster than most regulatory cysteines, implying that scavengers should outcompete signaling targets [107]. Redox relay models resolve this by positioning peroxiredoxins as signal receivers and transmitters. In this framework, PRDXs are oxidized by H_2_O_2_ to sulfenic intermediates and then transfer oxidizing equivalents to specific client proteins via transient protein–protein interactions and disulfide exchange, conferring both kinetic feasibility and target selectivity [108]. In mitochondria, PRDX3 coupled to thioredoxin 2 (Trx2) is central to matrix H_2_O_2_ handling, and its oxidation state, shaped by Trx2 recycling and NADPH supply, effectively encodes mitochondrial redox flux. When oxidant flux exceeds recycling capacity, PRDX hyperoxidation has been proposed to function as a “floodgate,” allowing H_2_O_2_ to escape high-affinity peroxidase buffering and engage broader, lower-affinity targets [109].

#### 3.1.6. Lipid-Derived Electrophiles: An Extended Signaling Repertoire

Beyond protein thiol oxidation, mitochondrial ROS can generate secondary electrophilic messengers through lipid peroxidation. Oxidation of polyunsaturated fatty acyl chains, particularly in cardiolipin-rich mitochondrial membranes, can yield reactive aldehydes such as 4-hydroxynonenal (4-HNE) and malondialdehyde (MDA) [110]. At high levels, these products form damaging adducts with proteins and nucleic acids; at low to moderate levels, however, electrophiles like 4-HNE can act as signaling mediators via Michael addition to nucleophilic residues, thereby activating the NRF2/KEAP1 pathway [ 111]. Cardiolipin oxidation also intersects apoptosis by weakening cytochrome c retention at the inner membrane [112]. Notably, several phytochemicals are electrophilic and converge mechanistically on KEAP1 sensor cysteines, conceptually aligning endogenous and exogenous mitohormetic cues [113] as discussed further below.

### 3.2. Sub-Mitochondrial Topology of mtROS Generation

Beyond chemical identity, the intramitochondrial site of mtROS generation is critical because it determines which redox systems, molecular targets, and cytosolic escape routes are accessible. Complexes I and III are the most frequently implicated ETC sources [114]. A key topological distinction is that Complex I primarily releases ROS into the matrix, whereas Complex III can release ROS into either the intermembrane space (IMS) or the matrix, depending on Q-cycle occupancy and respiratory state [115]. Redox-proteomic studies support this spatial specificity, showing that inhibitor-induced ROS at Complexes I and III preferentially oxidize matrix or IMS proteins, respectively [116,117].

Electron flow through the ETC converges on the ubiquinone pool. Electrons enter via Complex I or Complex II, reduce ubiquinone (Q) to ubiquinol (QH_2_), and are then transferred through Complex III and cytochrome c to Complex IV, where oxygen is reduced to water [118]. Proton pumping by Complexes I, III, and IV generates the electrochemical gradient that drives ATP synthase [119]. Within this architecture, Complex I can generate matrix-directed ROS from functionally distinct sites. At the flavin site (I_F_), elevated matrix NADH/NAD^+^ and constrained downstream electron flow favor ROS production driven by redox pressure and bioenergetic stalling. In the ubiquinone-binding region (I_Q_), a highly reduced Q pool can drive reverse electron transport (RET), producing matrix-directed superoxide, which has been linked to adaptive signaling in oxygen sensing, macrophage activation, myotube differentiation, and lifespan extension [120]. Thus, even within a single complex, the site and mode of ROS formation can yield distinct biological outcomes. Complex II indirectly shapes mtROS topology by modulating the QH_2_/Q ratio. Under high-succinate conditions or with restricted downstream electron flow, Complex II can generate ROS at its FAD site and promote Q-pool over-reduction, thereby favoring RET at Complex I and ROS production at Complex III. This is particularly relevant in succinate-rich contexts such as ischemia–reperfusion and inflammatory macrophage activation [121]. Complex III is topologically distinctive because superoxide generated at the outer Q_0_ site can be released into the IMS, where SOD1 converts it to H_2_O_2_ and enables communication with cytosolic redox targets via outer-membrane pathways such as VDAC. This IMS-directed route is central to hypoxia-associated HIF-1α stabilization, whereas matrix-directed Complex III ROS primarily engages matrix redox relays such as PRDX3/Trx2 [122].

Additional mitochondrial enzymes provide context-dependent ROS inputs that reflect tissue metabolism and substrate state. OGDH and PDH can produce ROS when NADH/NAD^+^ is high and substrate supply is excessive [123,124]; mG3PDH can release ROS toward the IMS, whereas electron transfer flavoprotein:ubiquinone oxidoreductase (ETF-QO) becomes important during high fatty-acid β-oxidation [125,126]. DHODH may contribute during proliferative states with high pyrimidine demand [127], and outer-membrane monoamine oxidases generate H_2_O_2_ during amine metabolism, particularly in the heart and brain [128]. These sources indicate that mtROS topology is determined not only by ETC electron leak but also by metabolic specialization.

Local redox microdomains further refine this topology. ROS-generating sites, SOD isoforms, PRDX/Trx systems, VDAC-containing outer-membrane interfaces, and cardiolipin-rich IMM regions can localize oxidant sources, signal converters, buffering enzymes, and redox-sensitive targets in close proximity. Such an organization may create functional signaling hubs that allow compartment-restricted ROS production to be selectively decoded rather than spreading as nonspecific oxidation [101,109,117,122].

ETC supramolecular organization adds a final layer of spatial control. Complexes I, III, and IV assemble into respiratory supercomplexes that modulate electron-transfer dynamics and Q-domain behavior. Supercomplex formation can reduce electron residence at leak-prone intermediates, whereas disruption by genetic perturbation, cardiolipin depletion, aging, or disease can elevate mtROS and alter release patterns [129,130]. Complex II is generally excluded from these assemblies and feeds electrons into the bulk Q pool, which can promote Q over-reduction under succinate-rich conditions [131,132]. Because cardiolipin supports supercomplex stability, changes in cardiolipin remodeling may influence mitochondrial ROS topology at the supramolecular level [133,134].

### 3.3. Reverse Electron Transport as a Distinct mtROS Signaling Mode

RET through Complex I is a condition-dependent source of mtROS that is mechanistically and thermodynamically distinct from forward-electron leak. Accumulating evidence places RET-derived ROS at the center of adaptive responses relevant to mitohormesis (reviewed in [135]).

#### 3.3.1. Thermodynamic Requirements and Experimental Discriminators

During forward transport, NADH reduces FMN at Complex I, and electrons traverse Fe–S centers to reduce Q at the I_Q_ region, thereby coupling proton pumping [136]. RET reverses this process as electrons from a highly reduced QH_2_ pool flow backward through Complex I toward FMN, reducing mitochondrial matrix NAD^+^ to NADH [137]. RET requires both a high QH_2_/Q ratio (e.g., robust succinate oxidation by Complex II with insufficient downstream re-oxidation) and a high proton-motive force (Δp; ΔΨm + ΔpH) (e.g., low ATP demand relative to substrate supply). When these conditions are met, the I_Q_ region becomes a major source of matrix-directed superoxide production [118]. Because the relevant RET-associated ROS-generating sites of Complex I face the matrix side of the IMM, RET-derived superoxide is primarily directed toward the matrix rather than released into the IMS. RET also intersects with pyridine-nucleotide metabolism: reverse electron flow reduces matrix NAD^+^ to NADH, and when the proton-motive force is sufficient, NADH can support NADPH regeneration via nicotinamide nucleotide transhydrogenase (NNT), thereby linking RET-derived ROS production to glutathione and thioredoxin recycling [118,138]. Two practical discriminators are used: rotenone, which blocks electron entry at the I_Q_ site and inhibits RET-ROS (though it can increase IF-linked ROS by stalling forward flow), and mild uncouplers, which lower ΔΨm and suppress RET-ROS. These pharmacological criteria are essential controls for attributing phenotypes to RET [60,138].

#### 3.3.2. Biological Contexts of RET-Derived Signaling

RET-ROS supports defined signaling roles across various settings, including carotid body oxygen sensing, where succinate-driven RET enhances mtROS that modulate ion channels in glomus cells, promoting depolarization and neurotransmitter release [139]. In macrophage activation, TCA cycle remodeling elevates succinate levels, and RET-ROS stabilizes HIF-1α, promoting IL-1β expression and linking metabolism to effector functions [59,140,141]. Elevating I_Q_/RET-ROS extends lifespan in *Drosophila*; this effect is abolished by AOX, which oxidizes QH_2_ and limits RET, indicating causality. During myogenesis, a transient mtROS pulse, partly mediated by RET, is required for myotube differentiation [60]. Additionally, ischemic preconditioning involves early reperfusion oxidizing accumulated succinate, creating a brief RET-ROS burst that activates protective pathways [142,143]. Collectively, these examples support RET as a regulated signaling mode with adaptive potential when activated in pulses and in a bounded manner, demonstrating its diverse roles in cellular processes.

#### 3.3.3. Pathological Escalation: Ischemia–Reperfusion Injury

The same mechanism becomes damaging when RET-ROS is excessive or prolonged. During ischemia–reperfusion, succinate accumulates (partly via reversed SDH). Reoxygenation and rapid re-energization drive large-scale RET, overwhelm matrix buffers, promote permeability transition, and trigger cell death. Limiting succinate accumulation (e.g., SDH inhibition) or preventing RET at reperfusion (e.g., reversible Complex I inhibitors) reduces infarct size in preclinical models [142,143]. This contrast illustrates the mitohormetic dose–time principle: identical biochemistry, yet divergent outcomes set by flux magnitude/duration relative to buffering capacity.

#### 3.3.4. Endogenous Control Points: Tuners of RET

RET is regulated by several interconnected control points. Succinate availability determines the extent of Complex II-mediated Q reduction; elevated succinate promotes RET, whereas limiting succinate oxidation attenuates it [142,144]. ΔΨm and Δp are particularly important because RET is highly sensitive to proton-motive force; mild uncoupling or increased ATP synthase flux can reduce RET-associated ROS while preserving ATP synthesis [118]. Supercomplex assembly may also influence RET by shaping Q-pool access: Complex I embedded within supercomplexes may experience a more directed Q environment, decreasing Q over-reduction, whereas free Complex I interacting with the bulk Q pool may be more prone to RET [145]. The NAD^+^/NADH ratio provides an additional constraint, as RET consumes NAD^+^; a low NAD^+^/NADH ratio limits further NAD^+^ reduction, whereas higher NAD^+^ availability can sustain RET flux, linking RET to broader metabolic and sirtuin-dependent pathways [83]. Finally, cardiolipin integrity stabilizes mitochondrial complexes and supercomplexes; cardiolipin oxidation can destabilize these structures and alter the local lipid environment around ROS-producing sites, potentially creating a feed-forward cycle of oxidative damage [146].

#### 3.3.5. Implications for Mitohormesis and Interventions

RET is attractive as a tunable mitohormetic node because it (i) arises only under specific thermodynamic conditions, namely high QH_2_/Q and high Δp; (ii) supports adaptive programs when transient and bounded, including oxygen sensing, immune activation, differentiation, and preconditioning; and (iii) can be modulated at multiple levels, including succinate metabolism, ΔΨm, Q-pool redox state, and NAD^+^ availability [83]. Accordingly, natural-biomolecule strategies that mildly reduce ΔΨm, modulate succinate handling, adjust Q-pool dynamics, or transiently shift NAD^+^/NADH may tune RET-derived ROS into a protective range without tipping the system into oxidative distress [147].

### 3.4. Redox Buffering Systems as Signal Shapers

The chemical identity (Section 3.1), sub-mitochondrial origin (Section 3.2), and electron-flow mode (Section 3.3) define the initial mtROS signal. The biological outcome then depends on mitochondrial and cytosolic buffering systems that shape signal amplitude, duration, spatial reach, and target selectivity. Because their capacity and regeneration rates set the threshold between oxidative eustress and distress, these systems are integral components of the signaling machinery rather than mere scavengers.

#### 3.4.1. Matrix Buffering: PRDX3/Trx2 and Glutathione Systems

The dominant H_2_O_2_-removing system in the mitochondrial matrix is the peroxiredoxin 3 (PRDX3)/thioredoxin 2 (Trx2)/thioredoxin reductase 2 (TrxR2) axis [109,148]. PRDX3 is a 2-Cys peroxiredoxin abundantly expressed in the matrix and can account for a large fraction of mitochondrial peroxidase activity in some cell types. PRDX3 reacts with H_2_O_2_ at near diffusion-limited rates (~10^7^ M^−1^ s^−1^), forms an intermolecular disulfide with its partner subunit, and is recycled by Trx2, which is restored by the selenoenzyme TrxR2 using NADPH [109]. The oxidation state of PRDX3 encodes local H_2_O_2_ flux relative to recycling capacity, enabling redox relay signaling to client proteins [149]. When H_2_O_2_ flux exceeds recycling capacity, PRDX3 can become hyperoxidized at its peroxidatic cysteine (Cᴾ–SO_2_H), transiently inactivating peroxidase activity and opening a “floodgate” that allows H_2_O_2_ to accumulate and reach lower-affinity targets. Repair by sulfiredoxin (Srx) is ATP-dependent and relatively slow, thereby imparting temporal memory to the mitochondrial redox response [150,151,152]. The floodgate threshold depends on PRDX3 abundance, Trx2/TrxR2 kinetics, and NADPH supply.

The glutathione system provides a parallel layer of matrix H_2_O_2_ buffering. Mitochondria do not synthesize glutathione (GSH) de novo and therefore rely on import from the cytosol via inner-membrane carriers [153,154]. The mitochondrial GSH pool is typically maintained in the millimolar range and can behave as a semi-autonomous compartment whose redox state reflects local mitochondrial conditions [155]. Within the matrix, glutathione peroxidases (GPx) reduce H_2_O_2_ and lipid hydroperoxides using GSH, generating oxidized glutathione (GSSG), which is recycled to GSH by glutathione reductase (GR) in an NADPH-dependent reaction [156].

GPx4 uniquely reduces phospholipid hydroperoxides, including oxidized cardiolipin, thereby helping preserve respiratory supercomplex stability (Section 3.2) and cytochrome c retention/apoptotic control (Section 3.1); GPx4 loss predisposes to ferroptosis [157,158]. Kinetically, PRDX3 dominates soluble H_2_O_2_ removal under typical conditions, whereas GPx enzymes, especially GPx4, become critical during high flux, PRDX3 hyperoxidation, or lipid peroxide burden [159]. The GSH/GSSG ratio integrates buffering status; sustained oxidation promotes protein S-glutathionylation, regulating targets such as Complex I, OGDH, and ANT, with potential feed-forward effects on mtROS under severe depletion [160].

#### 3.4.2. NADPH Supply and Δp Coupling

Matrix buffering by PRDX3/Trx2/TrxR2 and GSH/GPx/GR depends on NADPH. Thus, the mitochondrial NADPH regeneration rate is a primary determinant of matrix buffering capacity. Three principal sources contribute:

- Nicotinamide nucleotide transhydrogenase (NNT): an IMM enzyme that generates NADPH from NADH and NADP^+^, driven by proton-motive force [161,162]. A widely used caveat is the C57BL/6J *Nnt* loss-of-function variant, which can impair NADPH regeneration and influence redox phenotypes [163,164].
- Isocitrate dehydrogenase 2 (IDH2): produces NADPH while converting isocitrate to α-ketoglutarate, coupling TCA-cycle flux to buffering capacity. Oncogenic IDH mutations can alter NADPH homeostasis while generating 2-hydroxyglutarate, thereby reshaping redox states in cancer [165].
- Malic enzyme 3 (ME3): generates NADPH during malate-to-pyruvate conversion, with tissue-dependent contribution [166].

Because Δp drives NNT, NADPH buffering is coupled to the same bioenergetic variables that shape ROS production and RET. Conditions that reduce Δp, limit TCA-cycle flux, or impose simultaneous high demand on both Trx and GSH recycling pathways lower buffering reserve, reduce the PRDX3 floodgate threshold, and contract the eustress window, an effect proposed to become more prominent with aging [167].

#### 3.4.3. Cytosolic Decoding of IMS Signals

IMS-directed mtROS (Section 3.2) can reach the cytosol, where distinct buffering and relay networks govern propagation distance and target selectivity. PRDX1/2 serve as high-affinity sinks and relay hubs for pathways such as ASK1 and STAT3 [168]. Catalase, largely peroxisomal, provides high-capacity protection at elevated H_2_O_2_ rather than fine control in the low-signal range [169]. Cytosolic GSH/GPx1 adds further buffering capacity. Together, these systems create a spatial gradient: targets proximal to mitochondria experience higher effective H_2_O_2_ than those in the bulk cytosol, enabling selective signaling without global oxidation [170].

#### 3.4.4. Context and Mitohormetic Implications

The buffering systems described above do not operate uniformly across the organism. Their capacity, composition, and regeneration kinetics vary substantially with tissue type, subcellular location, age, and disease state, creating a heterogeneous landscape that is central to the context dependence of mitohormetic signaling. High-oxidative tissues such as the heart, brain, and skeletal muscle generally express robust antioxidant defenses, yet they may remain vulnerable because high metabolic flux requires correspondingly high buffering throughput, leaving a limited reserve margin [171]. Neurons present a particularly instructive case: they combine low catalase expression with PUFA-rich membranes susceptible to lipid peroxidation, resulting in a narrow eustress window [172,173]. Heterogeneity also exists within individual organs; for example, periportal and perivenous hepatocytes maintain distinct redox thresholds reflecting differences in oxygen tension and metabolic specialization [174,175]. Aging compounds this heterogeneity by progressively reducing PRDX3, Trx2, SOD2, and mitochondrial GSH levels while altering NADPH supply, collectively lowering the floodgate threshold and contracting the range of mtROS that can be safely buffered [176,177]. Disease states further remodel these networks in divergent directions: metabolic disorders can deplete mitochondrial GSH and favor peroxiredoxin hyperoxidation, whereas cancers often upregulate PRDX3/Trx2/NNT-linked buffering to tolerate elevated oxidant flux and resist ROS-inducing therapies [178].

This heterogeneity has several direct implications for the design and evaluation of mitohormesis-based interventions using natural biomolecules. First, buffering state influences pharmacology: the same mtROS-inducing input can be beneficial when buffers are intact but harmful when they are depleted. Second, NRF2 programs expand the eustress window by upregulating the thioredoxin and glutathione systems and NADPH-supporting enzymes, which can be as critical as modulating ROS production itself. Third, NADPH-centric strategies can target Δp/NNT coupling, IDH2/ME3 flux, or cytosolic NADPH production to shift buffering set points independently of ROS generation. Finally, combining mild mtROS stimuli with buffer-regeneration support may yield more consistent mitohormetic outcomes than either approach alone.

## 4. Natural Biomolecules as Context-Dependent Modulators of mtROS

Natural biomolecules, including plant-derived phytochemicals, fungal metabolites, marine compounds, microbiome-derived metabolites, and endogenous redox-active molecules, can influence mitochondrial electron transport, membrane potential, and redox signaling either directly or indirectly [179]. A central theme of this review is that many compounds historically classified as “antioxidants” on the basis of in vitro radical-scavenging assays exert biologically relevant effects primarily by modulating mitochondria-dependent signaling pathways rather than by directly neutralizing ROS [180]. These mechanisms may include transient, low-level pro-oxidative signals that activate adaptive stress-response pathways. This mechanistic reframing has important implications for experimental design, formulation, delivery strategies, and the translation of natural biomolecules into clinically meaningful interventions [180].

We first define the principal mechanistic categories of mitochondrial engagement and then evaluate major compound classes through the lens of context dependence.

### 4.1. Mechanistic Categories of Action

**Figure 2:**
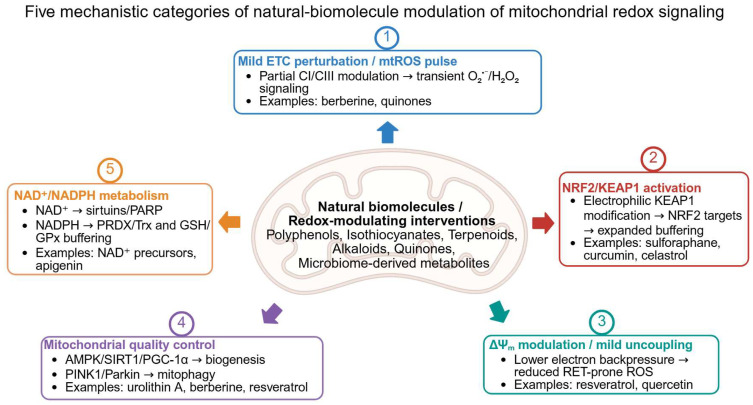
Mechanistic categories of natural biomolecules engaging mitochondrial redox signaling. Natural biomolecules and related redox-modulating interventions can engage mitochondrial ROS biology through five non-mutually exclusive mechanisms: mild ETC perturbation and transient mtROS pulses; electrophilic NRF2/KEAP1 activation; ΔΨm modulation and mild uncoupling; enhancement of mitochondrial quality control; and modulation of NAD^+^/NADPH metabolism. These mechanisms often overlap within the same compound and can yield adaptive or maladaptive outcomes depending on dose, timing, buffering capacity, metabolic state, topology, and disease context. Created in BioRender. Papaneophytou, C. (2026) https://BioRender.com/hlz8nn9 (accessed on 12 May 2026).

Before examining individual classes of compounds, it is useful to define the principal mechanisms by which natural biomolecules engage mitochondrial redox signaling. Based on current evidence, these mechanisms can be organized into five non-mutually exclusive categories (Figure 2).

The defining features of each category are summarized below.

- Category 1: Mild ETC perturbation and transient mtROS pulses. Certain compounds interact with components of the ETC, most commonly Complex I or Complex III, producing a brief, controlled increase in superoxide and/or H_2_O_2_ generation [181]. When moderate and reversible, this oxidant flux can engage adaptive mitohormetic programs, including NRF2 activation, UPR^mt^ induction, and mitophagy, without overwhelming buffering systems [182]. Representative examples include berberine, a partial Complex I inhibitor [183], and quinones that accept or shuttle electrons within the ETC [184].
- Category 2: Electrophilic activation of the NRF2/KEAP1 axis with mitochondrial coupling. Many phytochemicals are soft electrophiles that modify reactive KEAP1 cysteine residues, enabling NRF2 nuclear translocation and ARE-driven transcription [185,186]. NRF2 activation has a mitochondrial dimension because its target genes include redox-buffering and quality-control components, including PRDX3, Trx2, TrxR2, glutathione-related enzymes, NQO1, HO-1, and mitochondrial quality-control machinery [187]. By expanding buffering capacity, electrophilic NRF2 activators can raise the PRDX3 hyperoxidation threshold and widen the eustress window [188,189]. Sulforaphane [190], curcumin [191], and several terpenoids [192] prominently engage this mechanism.
- Category 3: Modulation of ΔΨm and mild uncoupling. Compounds that modestly reduce ΔΨm, either through weak protonophoric activity or endogenous uncoupling mechanisms, can attenuate excessive mtROS production, particularly RET-derived ROS, while maintaining adequate ATP output [57,193]. Several polyphenols, including resveratrol and quercetin, have been reported to exhibit mild uncoupling activity at low micromolar concentrations [194].
- Category 4: Enhancement of mitochondrial quality control. By activating regulators such as AMPK, SIRT1, and PGC-1α, some natural compounds promote mitophagy, mitochondrial biogenesis, and network remodeling [195]. Over time, this can shift the mitochondrial network toward higher efficiency and lower ROS production per unit of ATP. Resveratrol, berberine, and urolithin A are prominent examples acting through SIRT1-linked signaling, AMPK activation, and mitophagy induction, respectively [196,197].
- Category 5: Modulation of NAD^+^ metabolism and NADPH supply. Compounds that influence the NAD^+^/NADH ratio or NADPH availability affect mitohormesis by reshaping both mitochondrial signaling and buffering [198]. NAD^+^ precursors such as nicotinamide riboside and nicotinamide mononucleotide can support sirtuin and PARP activity and may contribute indirectly to NNT-linked NADPH regeneration [198,199]. Several plant-derived molecules also indirectly modulate NAD^+^ metabolism; for example, apigenin inhibits CD38, a major NAD^+^-consuming enzyme [200].

Most bioactive natural compounds engage more than one category (Table 2). This multi-target behavior should not be interpreted simply as nonspecificity; rather, it reflects the integrated nature of mitochondrial redox signaling and explains why compound effects depend strongly on dose, timing, metabolic state, and buffering capacity.

Although Section 4 focuses primarily on natural biomolecules, the clinical relevance of Category 2 is illustrated by omaveloxolone, a semi-synthetic oleanane triterpenoid and an NRF2 Pathway activator [231]. Omaveloxolone is approved for Friedreich ataxia in adults and adolescents aged 16 years and older, providing an important example of pharmacological modulation of redox pathways with clinical validation [232]. Its inclusion underscores that electrophilic activation of NRF2-linked adaptive programs can be therapeutically relevant, while also highlighting the need to distinguish clinically validated redox-modulating drugs from dietary or natural biomolecules whose efficacy is often supported primarily by preclinical or early human evidence [233,234].

### 4.2. Principal Classes of Natural Biomolecule Modulators

The main classes of natural biomolecules show how diverse compounds use limited mitochondrial redox-signaling mechanisms. Instead of exhaustive review, this section highlights examples and directs readers to Table 2 for detailed profiles.

Polyphenols and flavonoids are among the most extensively studied dietary phytochemicals with reported mitochondrial bioactivity [200,235]. This class includes stilbenes such as resveratrol, flavonols such as quercetin, flavanols such as EGCG, and curcuminoids such as curcumin. Despite structural diversity, many polyphenols contain redox-active catechol/galloyl motifs or electrophilic groups that enable redox cycling, thiol modification, and NRF2 engagement [236]. Resveratrol has been linked to AMPK/SIRT1/PGC-1α-associated mitochondrial biogenesis, fatty acid oxidation, autophagy/mitophagy, and stress-defense gene expression, although direct SIRT1 activation remains debated [201,237,238,239]. It may also modestly reduce ΔΨm and induce NRF2-dependent gene expression [194,240,241,242], but poor oral bioavailability limits straightforward clinical translation [243,244]. Quercetin similarly exhibits biphasic behavior: at low concentrations, it can support AMPK/PGC-1α-linked mitochondrial biogenesis, mild modulation of Complex I, and NRF2 signaling, whereas at higher concentrations, it can collapse Δψm, inhibit ATP synthase, deplete mitochondrial GSH, and induce apoptosis [203,204,245,246]. EGCG can inhibit Complex I and ATP synthase and activate AMPK/autophagy and NRF2-linked responses, but high-dose green tea extracts have been associated with rare cases of hepatotoxicity, underscoring dose- and exposure-dependent effects [205,247,248,249]. Curcumin, through its electrophilic α,β-unsaturated carbonyl groups, can activate NRF2/KEAP1 signaling and engage mitochondrial respiratory complexes, AMPK, SIRT1/PGC-1α, and PINK1/Parkin-associated mitophagy; however, poor bioavailability remains a major translational limitation [206,207,208,250,251,252].

Isothiocyanates provide a clearer example of electrophilic activation of NRF2/KEAP1. These compounds are generated from glucosinolates in cruciferous vegetables and readily react with nucleophilic thiolate ions [253]. Sulforaphane, produced from glucoraphanin, modifies KEAP1 sensor cysteines, classically Cys151, thereby enabling NRF2 nuclear translocation and ARE-driven transcription [209]. The mitochondrial relevance of sulforaphane lies in the NRF2-dependent expansion of glutathione and thioredoxin systems, which can support PRDX3/Trx2- and GSH/GPx/GR-dependent buffering and widen the eustress window [66]. Sulforaphane has also been linked to mitochondrial quality control and to transient increases in mtROS preceding NRF2 activation, consistent with a “trigger-then-buffer” model [182,254]. Translationally, sulforaphane is attractive because of its defined electrophilic chemistry, but internal exposure depends on food preparation, myrosinase activity, gut microbial conversion, formulation, and verification of NRF2 target engagement [255,256].

Terpenoids are structurally diverse natural products that often engage mitochondrial stress signaling, including electrophilic NRF2/KEAP1 activation, ΔΨm modulation, or mitochondrial quality-control effects [257,258]. Artemisinin derivatives illustrate the importance of chemical context: their endoperoxide bridge can undergo iron/heme-dependent activation, generating radical chemistry that may support adaptive signaling at low or transient exposure but promote oxidative distress in cells with high labile iron or limited buffering capacity [210,212,259,260]. Celastrol, a quinone methide triterpenoid, can modify KEAP1, inhibit HSP90, and alter mitochondrial bioenergetics, creating a narrow therapeutic window in which electrophilic/proteostatic signaling may become either adaptive or toxic depending on dose and buffering state [213,214,215,261,262,263,264].

Other terpenoids, including andrographolide, ursolic acid, and ginkgolide B, have been linked to NRF2 activation, AMPK/PGC-1α-associated mitochondrial biogenesis, preservation of ΔΨm, or PINK1-linked mitophagy in experimental models [75,216,217,218,219,265]. Overall, terpenoids reinforce the principle that dose, iron status, membrane environment, and redox-buffering capacity determine whether mitochondrial engagement remains hormetic or becomes damaging.

Alkaloids engage mitochondrial redox biology mainly through bioenergetic stress, AMPK activation, mitochondrial quality control, and, in some cases, NRF2 or NAD^+^/NADPH-linked mechanisms [266,267]. Berberine is the most prominent example: it partially inhibits Complex I, increases the AMP/ATP ratio sufficiently to activate AMPK, and has been linked to improved glucose metabolism, fatty acid oxidation, autophagy/mitophagy, and PINK1/Parkin-related quality control [183,220,221,268,269,270,271]. Its low oral bioavailability, despite reproducible metabolic effects, supports a possible gut-first mechanism involving intestinal exposure, microbiome-dependent metabolism, and bile-acid signaling. Caffeine has been associated with PGC-1α-dependent mitochondrial biogenesis and improved mitochondrial function, although its mitochondrial effects are difficult to separate from adenosine receptor antagonism and systemic neuroendocrine effects [222,272,273,274,275,276]. Piperine is most relevant as a bioavailability enhancer, particularly for curcumin, although it may also influence AMPK-linked metabolic pathways; its ability to alter drug metabolism and transporter function also raises interaction concerns [223,277,278].

Quinones and related redox-active compounds directly intersect mitochondrial electron flow because they can undergo reversible one- or two-electron reactions and influence Q-pool behavior [279,280]. CoQ10 is both an endogenous ETC component and a lipid-phase antioxidant in the inner mitochondrial membrane, where it supports electron transfer and protects membrane lipids, including cardiolipin [224,225,226,281]. In this framework, CoQ10 is best categorized as a Category 1-adjacent intervention that influences Q-pool electron transfer and the likelihood of mtROS formation, rather than as a traditional pro-hormetic stressor. Clinically, CoQ10 is relevant where deficiency or impaired Q-dependent electron transfer causes issues, like mitochondrial disorders, statin symptoms, and heart failure [282,283]. Thymoquinone illustrates biphasic quinone behavior: at low to moderate exposure, it can activate cytoprotective redox pathways, whereas at higher concentrations it can collapse ΔΨm, deplete GSH, drive oxidant flux beyond buffering capacity, and trigger apoptosis, particularly in tumor cells with limited residual buffering [227,284,285,286]. Paclitaxel is not a cytoprotective mitohormetic compound, but it is conceptually useful because its anticancer activity can involve mitochondrial ROS generation, mitochondrial permeability transition, ΔΨm loss, cytochrome c release, and caspase activation; resistance may also involve altered ROS handling and increased antioxidant capacity [228,229,230,287]. Thus, quinone-related redox biology can support either adaptive signaling or therapeutic induction of oxidative distress, depending on context.

The xenohormesis hypothesis provides an evolutionary rationale for why diverse natural biomolecules converge on conserved stress-response pathways [288]. Plants subjected to drought, UV radiation, pathogens, or nutrient limitations increase the levels of secondary metabolites such as polyphenols, terpenoids, alkaloids, and glucosinolate derivatives. When consumed, these molecules may act as external stress signals, activating pathways such as AMPK, sirtuins, NRF2, UPR^mt^/ISR, and mitophagy, overlapping with internal mitohormetic mechanisms. Electrophilic stress is a key point: many xenohormetic compounds, such as isothiocyanates, quinones, and α,β-unsaturated carbonyls, can modify KEAP1 sensor cysteines, similar to endogenous lipid electrophiles like 4-HNE [189,289]. Xenohormesis also helps explain the bioavailability paradox of compounds such as resveratrol and berberine, because local signaling in the gut or liver may induce mitokines or other endocrine-like mediators, including FGF21 and GDF15, even when systemic levels of the parent compound are low [290]. Microbiome metabolism adds another layer of context dependence; for example, urolithin A is produced from ellagitannins/ellagic acid in a microbiome-dependent manner and has been shown to induce mitophagy and improve mitochondrial function in preclinical models and early human studies [196,197,291]. Thus, dose, exposure pattern, microbiome metabolism, and systemic signaling determine whether natural biomolecules engage adaptive mitohormesis or shift vulnerable tissues toward distress.

## 5. Disease-Specific Contexts of Mitohormesis

The preceding sections established that the outcome of mtROS signaling is determined by the interplay of chemical species, sub-mitochondrial topology, temporal dynamics, buffering capacity, and metabolic state (Section 3), and that natural biomolecules can modulate this signaling at multiple nodes (Section 4). This section examines how these principles operate within specific disease contexts. A central theme is that the same mitohormetic mechanisms that promote cellular fitness in healthy tissues can become dysregulated, co-opted, or insufficient in disease states, where the eustress–distress threshold is often shifted by chronic inflammation, metabolic reprogramming, genetic vulnerability, or age-related decline in buffering capacity. Understanding these disease-specific contexts is essential for predicting when natural-biomolecule interventions are likely to engage beneficial adaptive programs and when they may risk exacerbating pathology. The discussion focuses on four disease contexts—neurodegeneration, metabolic disease, cardiovascular disease, and cancer—not because they exhaust the relevance of mitohormesis, but because they illustrate distinct ways in which disease biology reshapes the eustress–distress boundary: progressive buffering failure, nutrient-driven redox overload, acute and chronic mitochondrial stress, and tumor co-option of redox adaptation, respectively.

Table 3 summarizes the disease-specific mitohormetic landscapes discussed below, highlighting the dominant mitochondrial/redox disruptions, the direction of eustress–distress threshold shift, plausible intervention logic, representative natural biomolecules, and key translational caveats.

### 5.1. Neurodegeneration

Neurodegenerative diseases, including Alzheimer’s disease (AD), Parkinson’s disease (PD), amyotrophic lateral sclerosis (ALS), and Huntington’s disease (HD), share mitochondrial dysfunction as a convergent pathological feature despite differing in their primary molecular triggers [310]. The nervous system poses a uniquely challenging environment for mitohormetic signaling because neurons operate near the eustress–distress boundary even under basal conditions [311].

Several features explain this narrow neuronal eustress window. Neurons rely heavily on OxPhos and consume approximately 20% of total body oxygen despite representing only ~2% of body mass. Yet this high metabolic flux is coupled with limited antioxidant reserves, dependence on astrocyte-derived glutathione precursors, and PUFA-rich membranes vulnerable to lipid peroxidation [312]. Because neurons are post-mitotic, they cannot dilute damaged mitochondria through cell division and must maintain mitochondrial quality over decades through biogenesis, dynamics, transport, and mitophagy. Long axons further impose high demands on mitochondrial transport; disruption of this transport, as observed in AD, ALS, and HD, can cause local bioenergetic failure and increased mtROS generation at distal synapses [313]. Aging further contracts buffering capacity and reduces mitochondrial resilience, increasing the likelihood that otherwise adaptive redox signals cross into distress [314].

Disease-specific mechanisms reinforce this shift. In AD, amyloid-β oligomers impair mitochondrial respiration, interact with cyclophilin D at the mPTP, disrupt mitochondrial protein import, and elevate mtROS generation, while tau pathology impairs mitochondrial dynamics and axonal transport. APOE4 has also been linked to reduced mitochondrial respiratory efficiency [315,316]. Importantly, mitochondrial dysfunction is increasingly considered an early event in sporadic AD, potentially preceding overt amyloid pathology in at least some disease trajectories, which supports the rationale for early mitochondrial redox and quality-control interventions. In PD, PINK1 and Parkin regulate mitophagy, DJ-1 is a redox-sensitive mitochondrial protein, LRRK2 affects mitochondrial dynamics and calcium handling, and Complex I toxins such as MPTP and rotenone reproduce dopaminergic degeneration in experimental models [317,318]. In ALS, mutant SOD1 can accumulate in mitochondria and impair ETC function, while TDP-43 has been linked to altered expression of Complex I subunits [319,320].

These features suggest that the most plausible mitohormetic strategies in neurodegeneration are those that expand buffering capacity or improve mitochondrial quality control, rather than those that primarily generate additional mtROS. Sulforaphane, via NRF2 activation, has shown neuroprotective effects in models of AD, PD, and ALS [292]. Curcumin has shown protective effects in AD and PD models through NRF2 activation and mitochondrial biogenesis, although clinical translation is limited by poor bioavailability and blood–brain barrier penetration [294]. Urolithin A induces mitophagy and has shown benefit in *C. elegans* and murine AD models [293]. Caffeine is supported mainly by epidemiological associations with reduced PD risk and by preclinical evidence for mitochondrial biogenesis and mitophagy, although mitochondrial mechanisms are difficult to separate from adenosine receptor signaling [295].

Disease stage is a critical translational issue. In early or presymptomatic phases, when mitochondrial dysfunction is emerging but buffering reserve remains expandable, mitohormetic strategies may delay progression. In advanced disease, where mitochondrial networks are fragmented, redox buffering is exhausted, and neuroinflammation is established, the same interventions may be insufficient or destabilizing, particularly if they rely on Category 1 mtROS generation. This stage dependence argues for biomarker-guided stratification in clinical trials of natural biomolecules for neurodegeneration [321].

### 5.2. Metabolic Disease and Diabetes

Type 2 diabetes mellitus (T2DM), obesity, non-alcoholic fatty liver disease (NAFLD), and metabolic syndrome are interrelated conditions in which mitochondrial dysfunction and altered mtROS signaling act as both contributors to, and consequences of, disease progression. Unlike neurons, metabolic tissues such as liver, skeletal muscle, adipose tissue, and pancreatic β-cells generally possess substantial buffering capacity. However, chronic nutrient excess progressively depletes this reserve, leading to sustained oxidative stress that promotes insulin resistance, β-cell dysfunction, and hepatic steatosis [322].

Chronic caloric surplus drives pathological mtROS through converging mechanisms. Hyperglycemia increases glycolytic and TCA-cycle flux, elevates the matrix NADH/NAD^+^ ratio, promotes electron pressure at Complex I, and increases demand on NADPH-dependent antioxidant regeneration [323]. Excess fatty acid supply increases ETF-QO-mediated electron input into the Q pool and raises the QH_2_/Q ratio, favoring Q-pool over-reduction and Complex I ROS generation, including RET-derived matrix ROS, while incomplete fatty acid oxidation can generate acylcarnitines that further impair ETC function [324]. Succinate accumulation in obese adipose tissue and NAFLD hepatocytes may further promote Complex II-driven Q-pool over-reduction and RET, and elevated circulating succinate correlates with insulin resistance in T2DM [325]. Over time, sustained mtROS production depletes mitochondrial GSH, promotes PRDX3 hyperoxidation, reduces NAD^+^ availability through PARP activation, and establishes a feed-forward cycle of mitochondrial damage and further oxidant generation [58].

Pancreatic β-cells occupy a distinctive mitohormetic landscape because mitochondrial metabolism is central to glucose sensing. Glucose-stimulated increases in the ATP/ADP ratio trigger closure of K_ATP_ channels, membrane depolarization, Ca^2+^ influx, and insulin secretion. Moderate mtROS generation may contribute to this physiological signaling, whereas chronic hyperglycemia shifts the same system toward oxidative injury. Because β-cells express relatively low catalase and GPx1, they may be sensitive to H_2_O_2_ as a permissive signal for insulin secretion but vulnerable to sustained oxidative stress and apoptosis under diabetic conditions [326].

Skeletal muscle provides the clearest metabolic example of beneficial mitohormesis. Exercise transiently increases mitochondrial oxygen consumption and mtROS production, activating AMPK, PGC-1α, NRF2-dependent antioxidant programs, GLUT4 translocation, mitochondrial biogenesis, and improved insulin sensitivity. The mitohormetic nature of this response is supported by evidence that antioxidant vitamins C and E can blunt exercise-induced improvements in insulin sensitivity and mitochondrial biogenesis markers, indicating that mtROS signaling is necessary for full adaptation [327].

The metabolic disease context is favorable for natural-biomolecule interventions because target tissues are systemically accessible, buffering erosion may be reversible, and exercise and caloric restriction provide physiological precedents for mitohormesis. Berberine partially inhibits Complex I and activates AMPK, resembling an exercise-like bioenergetic stress; its glucose-lowering efficacy in T2DM is consistent with Category 1/Category 4 engagement [296]. Resveratrol shows context-dependent activity: in obese men, 30-day supplementation increased AMPK activity, elevated SIRT1 and PGC-1α protein levels, improved mitochondrial oxidative capacity, and enhanced insulin sensitivity, whereas mechanistic studies in muscle and C2C12 cells indicate that its effects are dose-dependent and not uniformly SIRT1-driven [297,298]. Sulforaphane has shown anti-diabetic effects in preclinical models and in a randomized trial of obese T2DM patients, in which broccoli sprout extract reduced fasting glucose and HbA1c, consistent with NRF2-mediated suppression of hepatic glucose production and buffering expansion [299]. Urolithin A, by inducing mitophagy and enhancing mitochondrial quality control, may help break the feed-forward cycle in which dysfunctional mitochondria generate excessive mtROS; early human data support improved muscle mitochondrial function in older sedentary adults [300].

### 5.3. Cardiovascular Disease

The heart is among the most mitochondria-dense organs, with mitochondria occupying approximately 30–40% of cardiomyocyte volume and supplying more than 95% of ATP required for continuous contractile activity. This dependence on OxPhos makes the myocardium both highly responsive to mitohormetic adaptation and highly vulnerable to mitochondrial distress [328].

Myocardial ischemia–reperfusion (IR) injury provides one of the clearest disease examples of the dual nature of mtROS. As discussed mechanistically in Section 3.3, ischemia promotes succinate accumulation, in part by reversing succinate dehydrogenase activity, along with adenine nucleotide depletion and altered mitochondrial membrane potential. Upon reperfusion, oxygen restoration and ETC re-energization drive rapid succinate oxidation and a burst of RET-derived superoxide at Complex I, contributing to mPTP opening, cytochrome c release, and cardiomyocyte death [142]. In contrast, ischemic preconditioning (IPC), in which brief ischemic episodes precede a sustained insult, represents a paradigmatic mitohormetic response. A controlled, transient ROS burst activates protective signaling pathways, including PKC-ε, mitochondrial ATP-sensitive potassium channels, NRF2, and HIF-1α, leading to improved calcium handling, enhanced antioxidant capacity, reduced mPTP sensitivity, and protection against subsequent prolonged IR [329]. Thus, the IPC paradigm demonstrates that cardioprotection versus cardiodestruction depends strongly on mtROS dose, duration, metabolic state, and buffering capacity.

In chronic heart failure (HF), sustained neurohormonal activation, pressure or volume overload, and substrate remodeling impose chronic stress on cardiomyocyte mitochondria. ETC complex activity declines, cardiolipin content and remodeling are disrupted, respiratory supercomplexes become destabilized, mitochondrial biogenesis and mitophagy are impaired, and CoQ10 levels may fall. Together, these changes shift the cardiomyocyte redox environment toward chronic distress, contributing to fibrosis, contractile dysfunction, and adverse remodeling [330].

Several natural-biomolecule interventions align mechanistically with this cardiovascular landscape. CoQ10 has one of the strongest clinical evidence bases among mitochondrial-targeted natural interventions: in the Q-SYMBIO trial, CoQ10 supplementation reduced major adverse cardiovascular events and cardiovascular mortality in chronic HF, consistent with restoration of Q-pool sufficiency, improved ETC efficiency, and enhanced IMM lipid antioxidant protection [301]. Resveratrol shows preclinical cardioprotection in IR, pressure overload, and doxorubicin-induced cardiotoxicity models through mechanisms involving SIRT1, AMPK, mild uncoupling, and NRF2 activation, although clinical translation remains inconsistent, likely reflecting bioavailability limitations and heterogeneity in baseline mitochondrial function [302]. Sulforaphane has shown cardioprotection in preclinical IR models, plausibly by expanding NRF2-dependent buffering capacity before injury and thereby mimicking aspects of late IPC [303]. Thymoquinone has shown preclinical benefit in IR and doxorubicin-induced cardiotoxicity models, with evidence for NRF2-mediated buffering expansion and preservation of ΔΨm and Complex I activity; however, clinical data remain limited [304,305].

### 5.4. Cancer: A Double-Edged Sword

Cancer represents one of the most complex disease contexts for mitohormesis. Tumor cells often exhibit elevated basal mtROS generation, altered mitochondrial metabolism, and expanded redox-buffering capacity, enabling them to tolerate oxidative pressure associated with oncogenic signaling, rapid proliferation, and metabolic rewiring. This creates a central paradox: mechanisms that protect normal cells from oxidative damage may also protect tumor cells from ROS-dependent death [331].

Cancer cells rewire mitochondrial metabolism to support biosynthesis and signaling rather than solely ATP production. Many tumors divert TCA-cycle intermediates toward lipogenesis, nucleotide synthesis, and redox balance, altering electron input into the ETC and reshaping mtROS production [332]. Mutations in SDH or fumarate hydratase lead to succinate or fumarate accumulation, stabilizing HIF-1α and modifying mtROS topology through altered Q-pool dynamics [333]. IDH1/2 mutations generate 2-hydroxyglutarate at the expense of NADPH, linking epigenetic dysregulation to altered redox-buffering capacity [334]. Complex III-derived mtROS have also been implicated in migration and metastasis through HIF-1α stabilization and matrix remodeling pathways, illustrating how mtROS topology can influence malignant behavior [335].

To survive elevated basal mtROS, many tumors upregulate redox-buffering machinery, including PRDX3, Trx2, TrxR2, glutathione biosynthesis, cystine import via xCT/SLC7A11, NADPH-generating pathways, and constitutive NRF2 signaling through KEAP1/NRF2 alterations or oncogene-driven activation. This expanded buffering allows tumor cells to maintain mtROS within a pro-survival range that supports proliferation, including through redox inhibition of PTP1B and PTEN and amplification of PI3K/Akt signaling, without crossing into apoptotic distress [336]. In this sense, cancer cells can co-opt mitohormetic machinery for survival and therapy resistance.

This co-opted landscape has direct implications for natural-biomolecule interventions. In cancer prevention, NRF2-activating compounds such as sulforaphane or curcumin may protect normal cells by expanding detoxification and redox-buffering capacity. In established tumors, however, the same buffering expansion could reinforce antioxidant defenses and contribute to chemoresistance, particularly in tumors with constitutive NRF2 activation; therefore, the prevention-versus-therapy distinction is critical [337]. Conversely, the distress arm of mtROS biology can be therapeutically exploited. Artemisinin derivatives generate iron-dependent radicals and may produce greater oxidative pressure in cancer cells with elevated labile iron or heme pools [306]. Paclitaxel-induced mitochondrial redox stress contributes to cytotoxicity, and resistance may intersect with redox-buffering logic because paclitaxel resistance has been associated with altered ROS dynamics, mitochondrial membrane potential, autophagy, and increased antioxidant capacity in tumor models [287,307]. High-dose quercetin can promote ΔΨm collapse, GSH depletion, and apoptosis in cancer models [308], while thymoquinone can similarly induce mitochondrial distress in tumor cells with limited residual buffering [309].

Thus, cancer requires a dual-phase interpretation of mitohormesis. Low-dose, buffering-expanding interventions may be most appropriate for prevention or normal-tissue protection, whereas treatment may require strategies that selectively overwhelm tumor redox buffering. This distinction demands careful dose optimization and patient stratification using biomarkers of oxidative vulnerability, including NRF2/KEAP1 status, GSH availability, SOD2 or PRDX3 expression, and mitochondrial dependence.

## 6. Translational Challenges and the Antioxidant Paradox

The mechanistic framework developed in Section 3, the natural-biomolecule profiles assembled in Section 4, and the disease-context analyses presented in Section 5 collectively support a compelling case for mitohormesis-based interventions. Yet clinical translation has been uneven: selected success stories, including CoQ10 in heart failure, berberine in T2DM, and sulforaphane for metabolic endpoints, coexist with a larger body of neutral or contradictory trial results, particularly for broadly acting “antioxidant” supplements. This section examines why this translational gap persists, why non-specific antioxidant strategies have often failed, and what a more rational, context-aware approach to mtROS modulation should look like.

### 6.1. Why Non-Specific Antioxidant Supplementation Fails

For decades, the oxidative-stress hypothesis of disease and aging predicted that exogenous antioxidants, including vitamins C and E, β-carotene, N-acetylcysteine, and selenium, should reduce oxidative damage, delay aging, and prevent chronic disease. This prediction was tested in numerous randomized controlled trials across cardiovascular disease, cancer, neurodegeneration, and all-cause mortality [338]. Overall, the results have been disappointing and, in some contexts, adverse.

Large clinical datasets illustrate this point. A Cochrane meta-analysis of 78 randomized trials involving 296,707 participants found no evidence that β-carotene, vitamin A, vitamin C, vitamin E, or selenium reduced all-cause mortality; β-carotene, vitamin A, and vitamin E were associated with increased mortality [339]. The SELECT trial reported that vitamin E did not reduce prostate cancer risk and was associated with a 17% increase in prostate cancer incidence [340]. In the ATBC trial, β-carotene increased lung cancer incidence and overall mortality in male smokers [341]. Similarly, the HOPE and HOPE-TOO trials found no cardiovascular benefit of vitamin E and suggested increased heart failure risk [342]. Finally, vitamin C and E supplementation during exercise training blunted improvements in insulin sensitivity and endogenous antioxidant responses, directly demonstrating that exogenous antioxidants can interfere with mitohormetic adaptation [48].

The mitohormetic framework explains why these outcomes are mechanistically plausible. First, non-specific scavenging can suppress adaptive eustress signals together with damaging oxidants. At physiological levels, mtROS activate NRF2, AMPK, UPR^mt^, mitophagy, and other adaptive pathways; broad ROS quenching may therefore reduce oxidation markers while impairing adaptive resilience [343]. Second, conventional antioxidants often operate with limited subcellular specificity. Vitamin C distributes primarily in aqueous compartments, whereas vitamin E partitions into membranes but is not selectively enriched in the mitochondrial matrix or cardiolipin-rich inner mitochondrial membrane domains. This compartmental mismatch may limit effects at key sites where mtROS are generated and decoded, including Complex I/III redox centers and matrix PRDX3/Trx2 and GSH/GPx systems [344]. Third, stoichiometric antioxidants cannot substitute for endogenous catalytic buffering systems such as PRDX3, GPx, TrxR2, and GR, which are continuously regenerated by NADPH-dependent pathways [345]. Fourth, antioxidant supplementation usually ignores context dependence. Trial populations were generally not stratified by mitochondrial function, redox-buffering capacity, age, disease stage, NADPH status, or baseline oxidative damage, even though the same intervention may be beneficial, neutral, or harmful depending on these variables [346]. Finally, some antioxidants can act as pro-oxidants under specific biochemical conditions: vitamin C can reduce Fe^3+^ or Cu^2+^ and potentially promote Fenton-type chemistry in metal-rich microenvironments, while α-tocopherol can form tocopheryl radicals if not efficiently regenerated [347]. Together, these mechanisms explain why non-specific antioxidant supplementation can fail, or even cause harm, despite the strong rationale behind the classical oxidative-stress model.

### 6.2. Toward Context-Aware Redox Interventions

The failure of non-specific antioxidants does not negate the therapeutic potential of redox modulation; rather, it indicates that redox interventions must account for biological context, temporal dynamics, and mitochondrial compartmentalization. The mitohormetic framework outlines several principles for rational intervention.

First, effective interventions should target the signal, rather than merely the ROS molecule. Rather than scavenging oxidants after they are produced, more rational strategies should modulate the source, timing, topology, or decoding of mtROS signals, or expand the buffering systems that determine their biological meaning. Natural biomolecules such as berberine, sulforaphane, and urolithin A illustrate this logic by engaging Complex I/AMPK signaling, NRF2-dependent buffering, and mitophagy, respectively.

Second, interventions should be matched to baseline buffering status. Category 1 compounds that mildly perturb the ETC are most likely to be beneficial when buffering capacity is intact or only moderately depleted, allowing a transient mtROS pulse to be decoded via PRDX/Trx and GSH-dependent signaling. In severely depleted systems, such as advanced neurodegeneration or end-stage heart failure, the same pulse may exceed the floodgate threshold and promote distress [108]. Conversely, NRF2 activators may be useful as preparatory or combination agents because they expand redox-buffering capacity and widen the hormetic window [348]. Pretreatment assessment of buffering status, using markers such as the GSH/GSSG ratio, peroxiredoxin hyperoxidation, thioredoxin-related markers, or NAD^+^ metabolomics, could support patient stratification and dose individualization [349].

Third, dosing should respect the temporal dimension of mitohormesis. Pulsatile or intermittent exposure is more likely to support adaptive remodeling than continuous exposure at the same average dose, because it preserves transient mtROS signaling while limiting chronic pathway activation. Exercise-timed administration of mild mitohormetic compounds is conceptually attractive, whereas co-administration with non-specific antioxidants may blunt exercise-induced adaptation [350]. In this context, pharmacokinetic features such as short half-life and peak–trough exposure may sometimes be advantageous because they preserve signal pulsatility.

Fourth, multi-category engagement should be leveraged rather than treated as non-specificity. Many natural biomolecules act at multiple nodes of the mitohormetic network. A compound or combination that generates a mild mtROS pulse, expands buffering capacity, and improves mitochondrial quality control may produce more durable adaptation than a single-target intervention. Such combinations remain hypotheses that require systematic testing of dose ratios, timing, and tissue-specific buffering status.

Finally, bioavailability and metabolic fate should be interpreted through a mitohormetic lens. Low systemic levels of parent compounds do not necessarily preclude biological activity if the compound acts locally in the gut or liver and triggers non-cell-autonomous signaling via mitokines, metabolites, or microbiome-dependent pathways. Microbiome-mediated bioactivation is also important; for example, ellagitannins can be converted into urolithin A, a mitophagy-inducing metabolite whose activity depends on individual metabotype [351]. Nevertheless, for diseases requiring direct target-organ exposure, such as neurodegeneration or myocardial injury, bioavailability remains a genuine constraint. Formulation strategies, prodrugs, bioavailability enhancers, and mitochondria-targeted delivery systems may therefore be necessary to translate mitohormetic mechanisms into reliable clinical effects.

### 6.3. Mitochondria-Targeted Redox Interventions

The compartmental mismatch problem, whereby conventional antioxidants act with limited subcellular specificity relative to the sites where mtROS are generated and decoded, has driven the development of mitochondria-targeted agents. Many of these compounds exploit the large negative membrane potential of the inner mitochondrial membrane (ΔΨm, approximately −150 to −180 mV) to accumulate within mitochondria. This strategy addresses one limitation of non-specific supplementation by delivering antioxidant, redox-modulating, or pro-hormetic payloads closer to compartments where mtROS production, buffering, and signaling are concentrated [352].

The most widely used targeting strategy conjugates bioactive molecules to lipophilic triphenylphosphonium (TPP^+^) cations. The delocalized positive charge of TPP^+^ enables membrane-potential-dependent mitochondrial accumulation, with matrix concentrations often estimated to be 100- to 1000-fold higher than cytosolic levels according to the Nernst relationship [353]. MitoQ, a TPP^+^-ubiquinone conjugate, is reduced to its ubiquinol form within mitochondria and can protect membrane lipids, including cardiolipin, from peroxidation. It has shown benefit in several preclinical models, although a Phase II Parkinson’s disease trial did not meet its primary endpoint despite demonstrating safety and tolerability; this negative result may reflect disease-stage limitations, insufficient target engagement, or disease-specific biology rather than simple failure of the targeting concept [344]. SkQ1, 10-(6′-plastoquinonyl)decyltriphenylphosphonium, is another TPP^+^-based mitochondria-targeted quinone developed by Skulachev’s group [354]. Although initially framed as a mitochondria-targeted antioxidant, some protective effects of SkQ-family compounds have also been linked to mild uncoupling and modulation of redox-dependent signaling pathways, consistent with the broader mitohormetic logic discussed here [355,356]. MitoTEMPO, a TPP^+^-piperidine nitroxide, functions as a mitochondria-targeted superoxide dismutase mimetic and may reduce superoxide-specific damage while preserving some H_2_O_2_-mediated signaling [357]. MitoSNO, a TPP^+^-S-nitrosothiol, transiently S-nitrosates Complex I during early reperfusion, slowing Complex I reactivation and reducing the RET-derived ROS burst that drives ischemia–reperfusion injury. This is mitohormetic in logic because it modulates the kinetics of ROS generation rather than simply scavenging ROS [358].

A complementary approach is represented by Szeto–Schiller (SS) peptides, including SS-31/elamipretide. Unlike TPP^+^ conjugates, SS peptides localize to the inner mitochondrial membrane primarily through interactions with cardiolipin rather than ΔΨm-dependent electrophoretic accumulation, which may preserve activity in depolarized or damaged mitochondria. SS-31 stabilizes cardiolipin–cytochrome c interactions, supports electron transfer, limits electron leakage, and protects cardiolipin from peroxidation, thereby helping preserve respiratory efficiency and membrane integrity [359]. Elamipretide has advanced into clinical testing in heart failure and primary mitochondrial myopathies, with mixed but informative results (reviewed in [360]).

Mitochondria-targeted natural-biomolecule conjugates extend this concept by linking known natural products to mitochondrial targeting moieties. MitoCurcumin has been reported to increase mitochondrial engagement and enhance cancer-cell cytotoxicity compared with unconjugated curcumin [361]. Mito-apigenin has shown enhanced mitochondria-directed anticancer activity in pancreatic cancer models [362]. These conjugates address bioavailability and compartmental mismatch simultaneously but introduce new challenges, including altered pharmacokinetics, potential mitochondrial overaccumulation, and dependence on ΔΨm for delivery. In severely depolarized mitochondria, TPP^+^-based compounds may accumulate less effectively precisely where intervention is most needed, a limitation that is partly addressed by Δψm-independent strategies such as SS peptides [363].

### 6.4. Redox-Based Biomarkers for Patient Stratification

A recurring theme throughout this review is that the efficacy and safety of mtROS-modulating interventions depend on baseline redox status, buffering capacity, metabolic state, and disease stage. Moving from population-level supplementation to precision redox medicine will therefore require clinically accessible biomarkers that capture these variables [364].

Several candidate biomarkers are relevant. The blood or erythrocyte GSH/GSSG ratio provides a systemic readout of glutathione redox status and may reflect aspects of cellular buffering reserve [365]. Plasma GDF15 and FGF21 can indicate mitochondrial stress in source tissues such as liver and muscle and may help determine whether mitochondrial stress-response pathways are already engaged [366]. Erythrocyte PRDX2 hyperoxidation may serve as a circulating reporter of peroxide exposure and systemic redox stress, although interpretation requires attention to erythrocyte-specific redox biology and turnover [367]. NAD^+^ metabolomics, including NAD^+^, NADH, nicotinamide, and related metabolites, can provide indirect information about the NAD^+^ pool available to support sirtuin activity, PARP-dependent repair, and NADPH-linked buffering pathways [368]. Classical oxidative damage markers, such as urinary 8-oxo-dG and F_2_-isoprostanes, remain useful when interpreted alongside buffering and mitokine markers because they help distinguish adaptive redox signaling from distress associated with macromolecular damage [369].

Validated biomarker panels integrating buffering status, mitohormetic pathway activation, mitochondrial stress signaling, and oxidative damage would substantially improve trial design. Such panels could enable patient stratification, dose individualization, and identification of disease stages in which mitohormetic interventions are most likely to be beneficial rather than neutral or harmful.

## 7. Future Directions

Despite substantial progress, several questions must be addressed before mitohormesis can move from a conceptual framework to a clinically operational paradigm.

First, the eustress–distress threshold remains difficult to quantify in vivo. Although this review has identified key determinants, ROS, sub-mitochondrial topology, electron-flow mode, buffering capacity, dose–time profile, and metabolic state, it is not yet possible to predict, for a given tissue or patient, which mtROS signal will be adaptive and which will be damaging. Progress will require quantitative models integrating real-time measurements of ΔΨm, compartment-specific H_2_O_2_ flux, PRDX oxidation state, GSH/GSSG ratio, NADPH availability, and downstream pathway activation.

Second, the physiological contribution of RET-derived ROS in humans remains incompletely defined. Evidence from model systems implicates RET in oxygen sensing, macrophage activation, myogenic differentiation, preconditioning, and lifespan regulation, but the extent to which RET operates as a regulated signaling mechanism in human tissues remains unresolved. Patient-derived cells, organoids, tissue-on-chip systems, and improved site-resolved redox reporters will be essential for determining when RET is adaptive, pathological, or therapeutically tunable.

Third, single-mitochondrion heterogeneity requires greater attention. Individual mitochondria within the same cell can differ in membrane potential, respiratory state, quality-control status, and likely mtROS output. Whether mitohormetic signaling reflects a population-average mitochondrial response or arises from spatially restricted signals generated by discrete mitochondrial subpopulations remains unclear. Super-resolution redox imaging and single-organelle functional assays could clarify how local mitochondrial signals are decoded at the cellular level.

Fourth, the role of the gut microbiome in systemic mitohormesis should be investigated more systematically. Microbial conversion of dietary precursors into bioactive metabolites, such as ellagitannins to urolithin A or daidzein to equol, may explain why some poorly bioavailable phytochemicals produce systemic effects. Inter-individual variation in microbiome composition and metabolite-producing capacity may therefore be a major determinant of the response to natural biomolecule interventions. Integrating metagenomics, metabolomics, and mitochondrial phenotyping into clinical studies will be essential.

Fifth, future work should determine whether mitokine signaling can be therapeutically harnessed. If mild mitochondrial stress in one tissue, such as the gut or liver, can induce FGF21, GDF15, humanin, MOTS-c, or related mediators that transmit adaptive signals systemically, then local mitohormetic activation could produce organism-wide benefits without requiring high systemic concentrations of the parent compound. However, the dose–response relationships, receptor mechanisms, tissue specificity, and risks of chronic mitokine elevation remain insufficiently understood.

Several methodological priorities follow from these unresolved questions. Site-specific and compartment-resolved mtROS reporters, including mitochondrial-targeted H_2_O_2_ sensors such as HyPer- and roGFP-based systems, should be adapted for physiologically relevant models, including organoids and tissue-on-chip platforms [370,371]. Redox reporting in natural-biomolecule studies should also be standardized, because many studies still rely on nonspecific probes, non-physiological concentrations, and incomplete characterization of baseline buffering status [372].

Preclinical studies should compare natural biomolecules using the five mechanistic categories defined in Section 4.1 across models with defined buffering capacity, age, disease state, and metabolic substrate conditions. Combination strategies should be systematically tested, particularly pairings combining a mild mtROS-generating stimulus with buffering expansion or mitochondrial quality-control support. Microbiome–mitochondria interactions should also be evaluated using defined microbial communities and standardized dietary precursor inputs.

Clinical translation will require biomarker-stratified trial designs. Trials of berberine in T2DM, sulforaphane in metabolic or neuroinflammatory endpoints, urolithin A in age-related mitochondrial decline, and CoQ10 in heart failure should incorporate baseline redox and mitochondrial stress biomarkers, including the GSH/GSSG ratio, PRDX hyperoxidation, plasma FGF21/GDF15, NAD^+^ metabolomics, and oxidative damage markers. Dosing studies should compare pulsatile versus continuous exposure, exercise-timed versus rest-timed administration, and fixed versus adaptive dosing schedules. In cancer, trials involving NRF2-activating compounds must distinguish prevention from treatment, because buffering expansion may protect normal cells but reinforce survival programs in established tumors with constitutive NRF2 activation.

Finally, technological development should focus on mitochondria-targeted delivery systems and disease-relevant human models. TPP^+^ conjugates, SS-peptide platforms, mitochondria-targeted natural-biomolecule derivatives, organ-on-chip models, and patient-derived organoids could help bridge the gap between mechanistic redox biology and clinically actionable intervention design.

## 8. Conclusions

Mitochondrial ROS are not intrinsically harmful or beneficial. Their biological meaning is determined by context: the chemical species produced, the sub-mitochondrial site of origin, the mode of electron flow, the temporal pattern of exposure, the capacity of local buffering systems, and the prevailing metabolic state. Together, these variables define a dynamic eustress–distress boundary that shifts with tissue type, age, disease state, and the timing of intervention.

Mitohormesis provides a coherent framework for understanding this biology. Low-to-moderate and transient mtROS signals can activate adaptive programs, including NRF2 signaling, AMPK and sirtuin pathways, UPR^mt^/ISR activation, mitophagy, and mitochondrial biogenesis. In contrast, excessive, sustained, or poorly buffered mtROS production promotes irreversible oxidation, lipid peroxidation, inflammation, permeability transition, and cell death. RET, redox relays, NADPH-dependent buffering, and mitochondrial quality control are therefore not peripheral details, but central determinants of whether mtROS signaling remains adaptive or becomes pathological.

This framework also reframes the biological actions of natural biomolecules. Many compounds historically described as “antioxidants” do not act primarily by stoichiometric radical scavenging at physiologically relevant exposures. Instead, polyphenols, isothiocyanates, terpenoids, alkaloids, quinones, microbiome-derived metabolites, and endogenous redox-active molecules often engage mitochondrial signaling through mild ETC perturbation, electrophilic activation of NRF2, modulation of ΔΨm, enhancement of mitochondrial quality control, or NAD^+^/NADPH-linked mechanisms. Their effects are therefore inherently context-dependent and frequently biphasic.

The inconsistent clinical evidence for antioxidant supplementation aligns with this view. Non-specific antioxidants can suppress adaptive eustress signals, act with limited subcellular specificity, fail to match endogenous catalytic buffering systems, and overlook inter-individual differences in redox state, disease stage, and mitochondrial function. The appropriate conclusion is not that redox-based interventions lack value, but that they must be redesigned around mitohormetic principles.

Disease context is decisive. In neurodegeneration, the narrow neuronal eustress window favors buffering expansion and mitochondrial quality control over direct mtROS-generating strategies. In metabolic disease, nutrient overload and buffering erosion create opportunities for exercise-mimetic, NRF2-activating, and mitophagy-inducing interventions. In cardiovascular disease, ischemic preconditioning provides mechanistic validation of mitohormesis, while CoQ10 provides one of the strongest clinical examples of mitochondrial redox support. In cancer, the same adaptive machinery can be co-opted by tumor cells, requiring a careful distinction between prevention, normal-tissue protection, and therapeutic induction of oxidative distress.

Future progress will depend on shifting from population-level antioxidant supplementation to context-aware, biomarker-guided, temporally optimized, and compartment-targeted redox modulation. The emerging tools—site-resolved ROS reporters, mitochondrial delivery platforms, redox biomarker panels, microbiome-informed metabolomics, and patient-derived models—make this transition increasingly feasible. The therapeutic goal is no longer simply to reduce ROS, but to tune mitochondrial redox signaling in the right direction, at the right time, in the right tissue, and for the right patient.

## Figures and Tables

**Table 1:** Key physicochemical and signaling features of mitochondrial ROS and ROS-derived electrophiles.

Species/ Messenger	Primary Mitochondrial Origin	Membrane Permeability/ Spatial Range	Dominant Chemistry /Targets	Signaling Competence (Typical)	Notes/Context Caveats	Ref.
Superoxide $(O_{2}^{\cdot -}$)	One-electron reduction of O_2_ at ETC redox centers (mainly Complex I/III)	Poor membrane permeability; compartment-restricted (matrix or IMS)	Reacts with Fe–S clusters (e.g., aconitase); precursor of H_2_O_2_ via dismutation	Indirect (mainly via conversion to H_2_O_2_)	Short-lived, typically microseconds to milliseconds depending on SOD activity; compartment-restricted local intermediate; can mobilize Fe–S cluster iron and amplify damage via downstream chemistry if excessive	[85,86,87]
Hydrogen peroxide (H_2_O_2_)	Dismutation of $O_{2}^{\cdot -}$ by SOD2 (matrix); SOD1 (IMS/cytosol); additional mitochondrial redox enzymes in some contexts	Moderately diffusible; transmembrane movement can be facilitated (e.g., aquaporins)	Reversible oxidation of low-pKₐ cysteines; redox relays via peroxiredoxins/thioredoxins	High (principal “information carrier”)	Longer-lived than superoxide, with effective persistence typically milliseconds to seconds depending on local peroxidase activity; principal diffusible redox signal; outcome depends on flux versus buffering	[54,88]
Hydroxyl Radical (HO**^·^**)	Secondary product via metal-catalyzed reactions (Fenton chemistry) from H_2_O_2_ in the presence of Fe^2+^	Extremely short range (near the site of generation)	Near-diffusion-limited reactions; largely indiscriminate damage to DNA, proteins, and lipids	Low (rarely selective signaling)	Extremely short-lived, typically nanoseconds; reacts near the site of formation; best interpreted as a mediator of oxidative distress rather than regulated signaling	[89,90]
Lipid-derived electrophiles (e.g., 4-HNE)	ROS-initiated lipid peroxidation (notably cardiolipin-rich membranes)	Diffusible within membranes and locally in cytosol; longer-lived than radicals	Michael addition to Cys/His/Lys can modify KEAP1 and other sensors	Moderate–high (dose-dependent)	Longer-lived secondary messengers than radicals; persistence depends on detoxification by glutathione conjugation, aldehyde dehydrogenases, and reductases; low/moderate levels can signal, whereas high levels form toxic adducts	[91,92]

Abbreviations: ETC, electron transport chain; Fe–S, iron–sulfur; GPx, glutathione peroxidase; GSH/GSSG, reduced/oxidized glutathione; HNE/4-HNE, 4-hydroxy-2-nonenal; IMS, intermembrane space; KEAP1, Kelch-like ECH-associated protein 1; NADPH, nicotinamide adenine dinucleotide phosphate (reduced); NRF2, nuclear factor erythroid 2–related factor 2; PRDX, peroxiredoxin; SOD, superoxide dismutase; Trx, thioredoxin.

**Table 2:** Mechanistic profiles of natural biomolecules targeting mitochondrial redox signaling.

Compound (Class)	Primary Mitochondrial Target(s)	Category *					Ref
		1	2	3	4	5	
Resveratrol (Stilbene)	Complex I (partial); SIRT1/AMPK axis	+	+/−	+	++	+	[201,202]
Quercetin (Flavonol)	Complex I (partial); ATP synthase	+	+	+/−	+	−	[203,204]
EGCG (Flavanol)	Complex I; ATP synthase; extracellular H_2_O_2_ generation	+	+	−	+	−	[205]
Curcumin (Curcuminoid)	Complexes I and II; KEAP1	+	++	−	+	−	[206,207,208]
Sulforaphane (Isothiocyanate)	KEAP1 (Cys151 primary); transient mtROS generation	+/−	+++	−	+	−	[209]
Artemisinin (Lactone)	Endoperoxide activation; Fe–S/heme chemistry; Complex I-linked stress	++	+	−	+/−	−	[210,211,212]
Celastrol (quinone methide)	KEAP1/NRF2; HSP90/TRAP1-linked proteostasis	−	+++	+/−	+/−	−	[213,214,215]
Andrographolide (Labdane diterpenoid)	KEAP1/NRF2-linked electrophilic signaling	−	++	−	+	−	[216]
Ursolic acid (Pentacyclic triterpene)	AMPK/PGC-1α axis	−	+/−	−	++	−	[217]
Ginkgolide B (Diterpene lactone)	Complex I support; ΔΨm preservation; PINK1/Parkin-linked signaling	−	+/−	−	++	−	[218,219]
Berberine (alkaloid)	Complex I (partial inhibition); AMPK axis	++	+	−	+	−	[183,220,221]
Caffeine (alkaloid)	PGC-1α axis; adenosine receptors	−	−	−	+	−	[222]
Piperine (alkaloid)	Bioavailability enhancer; AMPK-linked metabolic signaling	−	−	−	+/−	−	[223]
CoQ10 (Benzoquinone)	Q pool; ETC electron transfer; IMM lipid antioxidant	+/−	−	−	−	−	[224,225,226]
Thymoquinone (Benzoquinone)	Mitochondrial redox cycling; NRF2-linked signaling; ΔΨm/GSH disruption at high dose	+	+	+/−	−	−	[227]
Paclitaxel (taxane)	Microtubule stabilization; indirect mitochondrial dysfunction/mtROS; intrinsic apoptosis	+/−	−	−	−	−	[228,229,230]

* Categories: 1, mild ETC perturbation/mtROS pulse or ETC/Q-pool modulation; 2, electrophilic NRF2/KEAP1 activation; 3, ΔΨm modulation/mild uncoupling; 4, mitochondrial quality control, including biogenesis, dynamics, and mitophagy; 5, NAD^+^/NADPH modulation. Scoring key: (−) not reported or negligible; (+/−) weak, indirect, inconsistent, or context-dependent evidence; (+) moderate and documented; (++) strong and well characterized; (+++) primary or defining mechanism. Some compounds, such as CoQ10 and paclitaxel, are classified as category-adjacent because they modulate mitochondrial redox biology but are not classical pro-hormetic natural-biomolecule stressors. Cat., mechanistic category as defined in Section 4.1. Abbreviations: ETC, electron transport chain; IMM, inner mitochondrial membrane; NRF2, nuclear factor erythroid 2-related factor 2; ΔΨm, mitochondrial membrane potential; mtROS, mitochondrial reactive oxygen species.

**Table 3:** Disease-specific mitohormetic landscapes and intervention logic.

Disease Context	Dominant Mitochondrial/Redox Disruption	Eustress–Distress Shift	Most Plausible Intervention Logic	Representative Natural Biomolecules	Key Translational Caveat	Ref.
Neurodegeneration	Impaired mitophagy, axonal transport defects, low buffering reserve	Narrowed window; high risk of distress	Buffering expansion; mitochondrial quality control	Sulforaphane, urolithin A, curcumin, caffeine	Disease stage; BBB ^1^ penetration; advanced degeneration	[292,293,294,295]
Metabolic disease/ T2DM ^2^	Nutrient overload, high NADH/NAD^+^, Q-pool reduction, buffering erosion	Reversible chronic distress	Exercise-mimetic AMPK activation; NRF2; mitophagy	Berberine, sulforaphane, resveratrol, urolithin A	Baseline metabolic state; dose timing; gut-mediated effects	[296,297,298,299,300]
CVDs ^3^	IR-induced RET, cardiolipin loss, ETC ^4^ impairment, CoQ10 depletion	Acute RET ^5^ burst or chronic mitochondrial distress	Preconditioning mimetics; Q-pool support; NRF2 buffering	CoQ10, sulforaphane, resveratrol, thymoquinone	Timing relative to ischemia; HF ^5^ phenotype; formulation	[301,302,303,304,305]
Cancer	Elevated basal mtROS, expanded buffering, NRF2/KEAP1 alterations	Tumor cells co-opt eustress; therapy aims for distress	Prevention vs. treatment distinction; selective redox overload	Sulforaphane/ curcumin for prevention; artemisinin, paclitaxel, thymoquinone for distress	NRF2 status; GSH/SOD2/ PRDX3; tumor mitochondrial dependence	[287,306,307,308,309]

^1^ BBB: Blood–brain barrier; ^2^ T2DM: Type 2 diabetes mellitus; ^3^ CVDs: Cardiovascular diseases; ^4^ ETC: Electron transport chain; ^5^ HF: Heart failure.

## Data Availability

Not applicable.
